# Optimizing Inorganic Cs_4_CuSb_2_Cl_12_/Cs_2_TiI_6_ Dual-Absorber Solar Cells: SCAPS-1D Simulations and Machine Learning

**DOI:** 10.3390/nano15161245

**Published:** 2025-08-14

**Authors:** Xiangde Li, Yuming Fang, Jiang Zhao

**Affiliations:** 1 College of Integrated Circuit Science and Engineering, Nanjing University of Posts and Telecommunications, Nanjing 210023, China; b22030415@njupt.edu.cn (X.L.); fangym@njupt.edu.cn (Y.F.); 2 Nantong Institute, Nanjing University of Posts and Telecommunications, Nantong 226006, China

**Keywords:** perovskite solar cells, high power conversion efficiency, dual-absorber layer design, machine learning, eXtreme Gradient Boosting, SHapley Additive exPlanations

## Abstract

Perovskite solar cells (PSCs) have emerged as a promising contender in photovoltaics, owing to their rapidly advancing power conversion efficiencies (PCEs) and compatibility with low-temperature solution processing techniques. Single-junction architectures reveal inherent limitations imposed by the Shockley–Queisser (SQ) limit, motivating adoption of a dual-absorber structure comprising Cs_4_CuSb_2_Cl_12_ (CCSC) and Cs_2_TiI_6_ (CTI)—lead-free perovskite derivatives valued for environmental benignity and intrinsic stability. Comprehensive theoretical screening of 26 electron/hole transport layer (ETL/HTL) candidates identified SrTiO_3_ (STO) and CuSCN as optimal charge transport materials, producing an initial simulated PCE of 16.27%. Subsequent theoretical optimization of key parameters—including bulk and interface defect densities, band gap, layer thickness, and electrode materials—culminated in a simulated PCE of 30.86%. Incorporating quantifiable practical constraints, including radiative recombination, resistance, and FTO reflection, revised simulated efficiency to 26.60%, while qualitative analysis of additional factors follows later. Furthermore, comparing multiple algorithms within this theoretical framework demonstrated eXtreme Gradient Boosting (XGBoost) possesses superior predictive capability, identifying CTI defect density as the dominant impact on PCE—thereby underscoring its critical role in analogous architectures and offering optimization guidance for experimental studies. Collectively, this theoretical research delineates a viable pathway toward developing stable, environmentally sustainable PSCs with high properties.

## 1. Introduction

The accelerated pace of industrialization has driven fundamental structural shifts within the global energy supply and demand framework [1]. While historically dominant, traditional carbon-based energy systems confront dual challenges: imminent resource depletion and environmental degradation caused by greenhouse gases and harmful combustion byproducts [2]. These intertwined pressures have compelled the academic community to urgently advance towards renewable energy systems that are both efficient and environmentally benign. As the pivotal field of renewable energy, photovoltaic technology plays a crucial role in promoting energy transformation [3], whose technological iteration is directly linked to the progress towards achieving carbon neutrality [4]. Despite the widespread market dominance of crystalline silicon photovoltaic devices [5], their production process, which relies on high-purity silicon refinement and energy-intensive high-temperature treatment, makes for elevated costs [6]. Furthermore, inherent material limitations constrain the PCE, spawning a new generation of a photovoltaic material research boom denoted by perovskite, which can be synthesized by low-cost solution-based methods [7]. Simultaneously, perovskite materials exhibit exceptional optoelectronic properties, including remarkable carrier mobility and favorable light absorption coefficients. Critically, their tunable band gap enables novel laminated battery designs and expanded solar radiation utilization [8]. Notably, Kojima et al. demonstrated a PSC based on CH_3_NH_3_PbI_3_ in 2009, achieving a PCE of 3.8% [9]. By 2022, Tockhorn et al. had elevated the PCE of a laminated battery to an amazing 29.8% through nanotextured interface technology [10], which underscores the transformative potential of PSCs beyond existing routes. Nevertheless, lead, as the core component of cutting-edge PSCs, raises significant challenges owing to toxicity and environmental compatibility [11]. The migration risk of heavy metal components through the device life cycle not only diminishes PCE but also poses substantial hurdles to commercialization [12]. Consequently, researchers are tasked with addressing the environmental compatibility of materials while simultaneously improving efficiency.

Belonging to the double perovskite cubic crystal system, CCSC features a three-dimensional lattice structure composed of [CuCl_6_] and [SbCl_3_] polyhedrons. Robust bonding interactions of chlorine-based ligands and the charge balance provided by copper-antimony ions maintain the stability of the material [13] while inhibiting ion migration and lattice distortion. Further, lead-free components contribute to its minimal toxicity, with copper and antimony elements providing significantly lower environmental risks compared to traditional lead-based systems. Meanwhile, the relatively narrow band gap guarantees its potential as an absorber layer material [14]. Although the high electron effective mass inherent to CCSC bulk may result in reduced electron mobility [15], Wang et al. applied ultrasonic stripping technology to modify CCSC nanocrystals (CCSCNCs) into direct band gaps, while diminishing the electrons’ effective mass and speeding up the photoelectric response [16]. Numerous studies have investigated CCSC as an absorber layer. He et al. conducted a simulation on the structure of Fluorine-doped Tin Oxide (FTO)/TiO_2_/CCSCNCs/Cu_2_O/Au through SCAPS-1D, realizing 23.07% PCE, which demonstrated the significant potential of CCSC as an inorganic PSC absorber layer [17]. Subsequently, Yadav et al. developed a PSC incorporating CCSC material, systematically exploring the impact of HTL presence or absence. Their findings illuminated that FTO/SnO_2_/CCSC/CuSCN/Au and FTO/SnO_2_/CCSC/Pt configurations achieved breakthrough PCEs of 29.71% and 29.69%, respectively [2], which offered a paradigm for the optimization of PSC structures. More recently, Khan et al. introduced a CCSC quantum dot interface layer at the absorber layer/HTL in CsGeI_3_-based PSC. This modification successfully enhances PCE and short circuit current (J_sc_), further broadening the applicability of CCSC [18].

Cs_2_TiX_6_ (X = Cl, Br, I) exhibits a perovskite-like cubic structural framework, characterized by a three-dimensional network of [TiX_6_] octahedrons connected by sharing vertices, with cesium ions located at cubic gap sites [19,20]. The strong coordination bonds between titanium and halogens minimize the tendency for structural distortion, while the purely inorganic nature eliminates the risk of thermal degradation often associated with organic components. Moreover, the absence of lead ensures minimal hazard to the environment. As the absorber layer of PSCs, the material demonstrates excellent properties on account of its tunable band gap and alignment with the solar spectrum, while the regulation of halogen allows for the optimization of the carrier transport path. Through the three-dimensional rigid structure, the material inhibits the movement of ions and the defect concentration at the grain boundary, while the chemical inertness of halogens enhances the stability of the material under varying light conditions [21]. Considerable research has investigated Cs_2_TiX_6_. Previously, Chen et al. utilized a low-temperature gas-phase method to synthesize Cs_2_TiBr_6_ thin films, with the PSC that was fabricated by this method achieving 3.3% PCE. This research laid the foundation for the optimization of Ti-based materials [22]. Later, Ahmad et al. optimized the structure of Indium tin oxide (ITO)/TiO_2_/CTI/CdTe/Au to reach a PCE of 15.06%, which fully demonstrated the prospect of CTI material as an absorber layer [23]. In the framework of SCAPS-1D, Moiz et al. compared the properties of three device configurations: ITO/Nb_2_O_5_/Cs_2_TiX_6_/PEDOT:PSS/Au, while the structure with CTI attained a maximum PCE of 18.5% [24]. In summary, this study focuses on CTI material investigation.

ML has found extensive application in the field of PSC optimization, primarily for tuning the parameters of various layers to enhance PCE. Typical employed optimization algorithms include XGBoost and random forest (RF). Previously, Kenfack et al. integrated XGBoost with a polynomial regression algorithm to achieve an R^2^ exceeding 0.99 while simultaneously reaching a PCE of nearly 30%, which surpasses other earlier findings for the same absorber layer [25]. Meanwhile, Kaur et al. employed multiple methods, including RF and XGBoost, for comparison, ultimately achieving a notable rise in PCE from 9.5% to 21.9% [26]. In recent studies, ML has increasingly been utilized to identify the most influential variables. This trend arises as, although various optimization algorithms can elevate PCE with superior R^2^, the optimization outputs often represent highly precise decimal values [27], which is challenging to replicate in the practical fabrication process. However, if parameters are forcibly constrained to integers, the search space for high-dimensional parameters will collapse from the continuous infinite field to the finite discrete points, which enables exhaustive searches through brute force, with the significance of high-order optimization algorithms lost. What is more, the optimization results require extensive parameter adjustment, with their limited role in the experimental settings. On the contrary, it is more practical to rank the significance of variables by decision trees or linear regression (LR) models. Numerous examples have demonstrated the effectiveness of this approach. For instance, Uddin et al. achieved an R^2^ of 0.7 based on ridge regression, identifying defect density as the most crucial factor affecting PCE [28]. Similarly, Ghost et al. utilized the RF model to evaluate multiple property parameters, judging thickness as the most significant factor under conditions of superior R^2^ [29]. Further, Maoucha et al. employed the enhanced RF model for regression analysis, concluding that the interface defect concentration of HTL/absorber layer had the foremost impact on the properties [30]. Overall, these achievements have all provided guidance for experiments.

Although single-junction solar cells exhibit continuously improving efficiencies, their theoretical performance remains confined by the SQ limit [31]. Currently, constructing double or multi-layer heterojunctions represents a promising strategy to surpass the SQ constraint. A typical dual-absorber layer heterojunction, formed by vertically integrating wide-bandgap and narrow-bandgap perovskite materials, amplifies full-spectrum light harvesting through spectral-response complementarity. This enhancement significantly boosts carrier generation and photoelectric conversion efficiency (PCE) [32]. Building on this concept, our study employs a dual-absorber layer configuration, specifically targeting its structural optimization. This work introduced an innovative approach by combining CCSC and CTI, proposing the previously unreported Glass/FTO/ETL/CTI/CCSC/HTL/Au architecture. This dual-absorber design demonstrates unique synergistic benefits: band gap complementarity between CCSC and CTI minimizes photon loss, while the heterojunction’s energy-band gradient facilitates directional carrier transport, efficiently suppressing interface recombination [33]. Following compatibility assessments of 26 ETLs and HTLs based on efficiency and band alignment, STO and CuSCN were ultimately selected. Subsequent absorber layer optimization achieved a notable PCE of 30.87%. After evaluating multiple factors, Cu-doped C replaced the back electrode material. Further efficiency corrections accounting for radiative recombination, resistance, and FTO reflection yielded a revised PCE of 26.60%. Analysis of non-ideal factors also identifies challenges for practical implementation, while temperature-dependent performance tests demonstrate the PSC’s exceptional stability.

Among the aforementioned algorithms, LR, RF, and XGBoost were selected for comparative PSC modeling. Evaluation employed two metrics: the coefficient of determination (R^2^) and root mean square error (RMSE), with XGBoost exhibiting superior performance. SHapley Additive exPlanation (SHAP) analysis subsequently ranked the significance of independent variables in the XGBoost model, revealing that CTI/CCSC interface defect density dominates open-circuit voltage (V_oc_) influence, whereas CTI defect density emerged as the most significant factor for J_sc_, filling factor (FF), and PCE. This insight provides directional guidance for optimizing analogous structures.

## 2. Methods

### 2.1. SCAPS-1D Simulation

SCAPS-1D (version 3312) [34] is a capable numerical simulation platform for PSCs, encompassing the governing equations for continuity, transport mechanics, and Poisson. It enables optimization of heterostructure configurations, investigation of defect mechanisms, prediction of environmental responses, and validation of novel structures. Due to its high computational efficiency and extensive database, SCAPS-1D finds widespread use in the parametric modeling of PSCs. Further details are provided in Section SA of Supporting Information.

**Figure 1:**
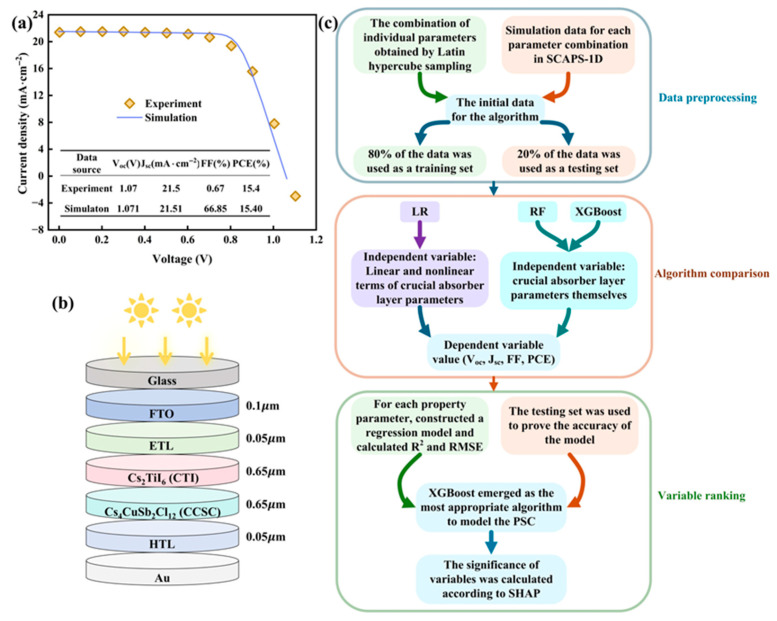
(**a**) SCAPS-1D verification; (**b**) device architecture diagram; (**c**) ML application flow.

Before simulations, the FTO/TiO_2_/CH_3_NH_3_PbI_3_/spiro-OMeTAD/Ag PSC structure validates SCAPS-1D [35], as revealed in Figure 1a. The performance confirms the software’s capability, with all the input data provided in Section SB of Supporting Information.

The device structure adopted comprises Glass/FTO/ETL/CTI/CCSC/HTL/Au, presented schematically in Figure 1b. For ETL and HTL, 13 candidate materials were screened for each layer. Input data for all layers and interfaces are provided in Section SC of Supporting Information.

The final PCEs were derived under the following assumptions: with exclusions applied to optical effects (such as interfacial reflections), non-radiative recombination mechanisms beyond Shockley–Read–Hall (SRH) recombination, interface issues (e.g., lateral inhomogeneity and interface roughness), and environmental fluctuations (including temperature and humidity variations). However, most of these assumptions can only be qualitatively discussed in Section 3.12 due to the limitations of SCAPS-1D.

### 2.2. Machine Learning

ML, a core component of AI, establishes models through the construction of mapping relationships between data and target values for prediction. Advances in chip computing power and the advent of the big data era have enabled widespread application of this technology across diverse fields. Meanwhile, ML addresses inefficiencies inherent in traditional methods, substantially shortening research and development timelines [36].

Within PSC research, ML demonstrates significant technical value for analyzing efficiency-determining factors and property attenuation. Therefore, LR and tree-based methods were selected from mainstream ML approaches.

Operating on the assumption of linear feature–target relationships, LR identifies optimal hyperplanes for continuous value prediction while allowing limited nonlinearity through adding an independent variable [37]. Thus, LR is characterized by simplicity and interpretability.

To address linear models’ limitations in complex nonlinear contexts and single decision trees’ overfitting tendency, RF constructs numerous trees whose predictions are aggregated [38]. Introducing randomness enhances robustness and generalization, typically outperforming singular models.

Advancing beyond traditional ensembles, XGBoost’s gradient boosting framework sequentially trains decision trees to correct predecessor residuals. Combining optimized algorithms, regularization, and parallel processing, it surpasses methods like RF in accuracy/speed efficiency, establishing itself as a premier tool for complex regression challenges [39].

Model interpretation employs the SHAP algorithm, grounded in the Shapley value from game theory. This approach performs additive decomposition of prediction results by computing the conditional expected contribution of features across varying subset combinations. Its core mechanism establishes the expected output of the data distribution as the baseline, quantifying the directional offset each feature induces relative to this baseline for a single prediction. Crucially, the method ensures local consistency: where the sum of all feature contributions equals the difference between the predicted value and the baseline. Through perturbation sampling, this technique enables both the interpretation of individual decision paths and the revelation of interaction mechanisms among high-dimensional features.

MATLAB R2024b and Python 3.11 were selected to construct these ML models owing to their robust numerical capabilities, with Figure 1c depicting the implementation process employed by these algorithms.

## 3. Findings and Analysis

### 3.1. Choice of ETL and HTL

**Figure 2:**
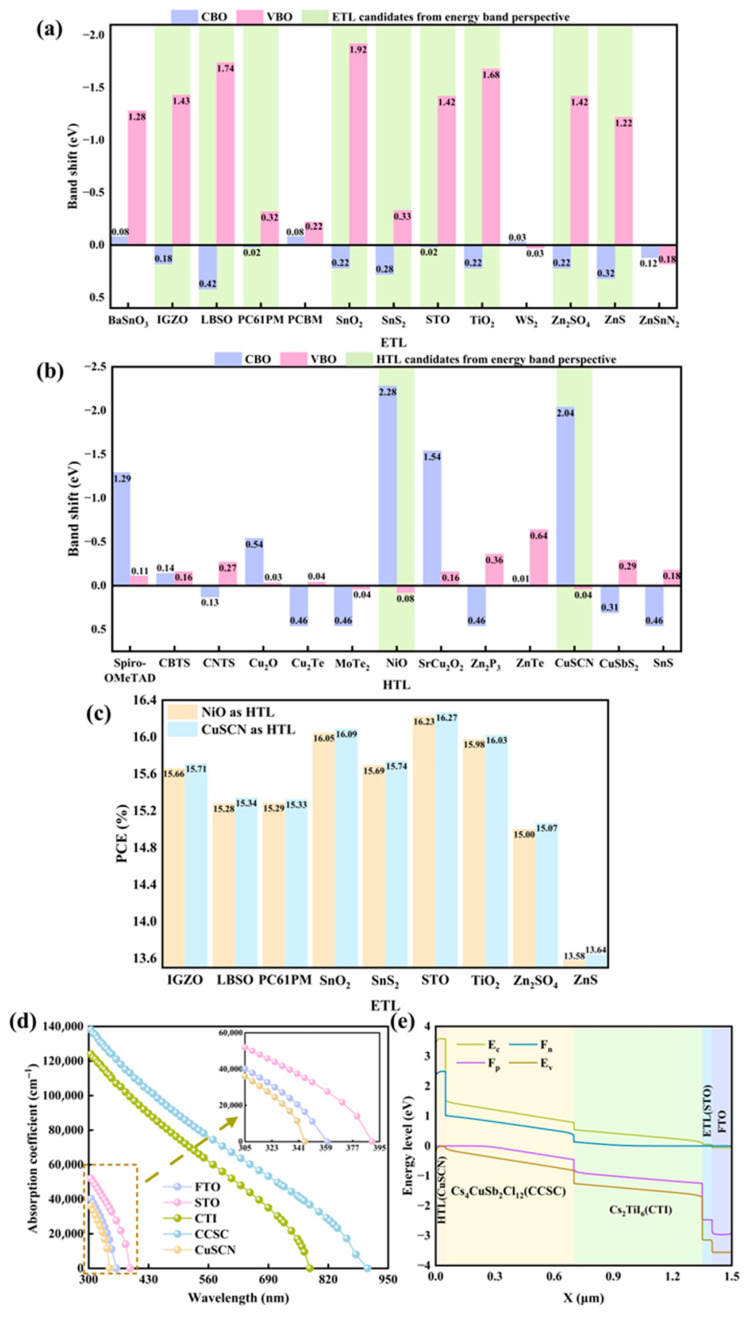
(**a**) CBO and VBO parameters across multiple ETL materials; (**b**) CBO and VBO parameters across multiple HTL materials; (**c**) the efficiency value of the selected ETL and HTL combination; (**d**) the absorption spectrum of the device; (**e**) initial energy band diagram of equipment.

This study explores optimal charge transport layer material selection from multiple dimensions. Within heterojunction energy band engineering, carrier transport is precisely modulated by energy band alignment. For ETL, a positive CBO significantly enhances electron migration efficiency while establishing a carrier injection barrier gradient. Simultaneously, a negative VBO effectively blocks hole reverse propagation. Similarly, in HTL systems, a positive VBO creates a stepped hole injection channel, whereas a negative CBO inhibits electron reverse diffusion. Figure 2a,b present VBO and CBO values for various ETL and HTL materials (determined by Equation (1), where *E_c_* and *E_v_* denote conduction band level and valence band level, respectively), highlighting in green those charge transport layer materials exhibiting optimal band alignment with the absorber layer. As Figure 2c illustrates, the 14 ETL/HTL pairings constructed from these materials display PCE variations, providing the rationale for selecting STO as ETL and CuSCN as HTL. Subsequently, using HTL material as an example, Table 1 further validates interface compatibility for the selected material within the designed PSC via lattice matching analysis (given by Equation (2), where *a_s_* and *a_e_* represent substrate and epitaxial layer lattice constants, respectively) [7].

Based on the designed PSC structure, the initial PCE reaches 16.27%, with absorption spectra shown in Figure 2d. The absorption coefficient is defined by Equation (3), where *h* and *c* signify Planck’s constant and light speed, while λ and *E_g_* denote light wavelength and absorber layer band gap, respectively. Functioning as a transparent conductive electrode, FTO maintains low absorption across the visible spectrum, ensuring efficient light transmission to the absorber layer. The primary role of ETL and HTL involves carrier extraction and transport, which contributes to their relatively weak visible light absorption. Given the strong dependence of the absorption coefficient on the material band gap, CCSC exhibits superior absorption to CTI throughout the full wavelength range. Figure 2e depicts the band alignment diagram for the constructed PSC, where the device’s gradient band structure constitutes an interface barrier reducing carrier transmission resistance.

(1) $$\begin{array}{c} CBO=E_{C,ETL/HTL}-E_{C,CTI/CCSC}\\ VBO=E_{V,CTI/CCSC}-E_{V,ETL/HTL} \end{array}$$

(2) $$\delta =\frac{2\left|a_{s}-a_{e}\right|}{a_{s}+a_{e}}\times 100\%$$

(3) $$\alpha =(\alpha_{0}+\frac{\beta_{0}\lambda }{hc})\sqrt{\frac{hc}{\lambda }-E_{g}}$$

### 3.2. Impact of Defect Density of Absorber Layer on Properties

The defect density of the absorber layer directly impacts carrier dynamics, thereby altering the overall properties of PSC. In the CTI layer, increased deep-level imperfections resulting from high defect density hinder the directional migration of photo-generated carriers and promote bulk phase recombination of electron–hole pairs. This condition contributes to a marked reduction in carrier lifetime, ultimately diminishing V_oc_. Concurrently, defects within the CCSC layer establish localized barriers impeding carrier transport, thereby exacerbating non-radiative recombination and lowering J_sc_. Furthermore, the synergistic effect of defects across the dual-absorber layer becomes more pronounced. Carrier retention caused by the CTI layer defects amplifies the imbalance of the carrier concentration gradient in CCSC, exhibiting a cross-layer recombination phenomenon that induces the concurrent decline of V_oc_ and FF [40].

**Figure 3:**
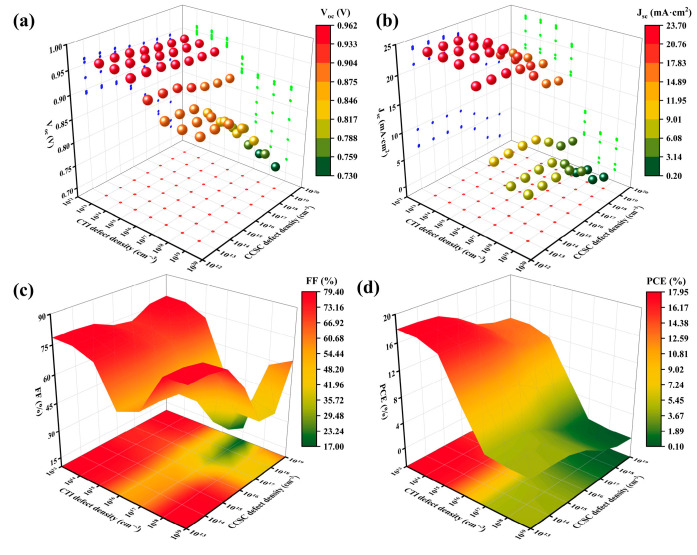
Impact of absorber layer defect density on PSC properties: (**a**–**d**) influence on V_oc_, J_sc_, FF, and PCE.

Figure 3a–d illustrates the impact on PSC properties as the absorber layer’s defect density ranges from 10^13^ cm^−3^ to 10^19^ cm^−3^. Variations in PCE exhibit a relatively regular pattern, while the non-monotonic behavior of FF stems from competing factors within the material. On the one hand, bulk defects in CTI may generate shallow-level defects, which, at appropriate concentrations, facilitate carrier migration and thereby enhance FF. Conversely, deep-level recombination centers formed by high defect density suppress FF. On the other hand, bulk defects in CCSC can modulate the degree of local band bending. An appropriate density promotes carrier separation, whereas an excess establishes a potential barrier. This phenomenon demonstrates the dualistic impact on FF [41]. Thus, these competing mechanisms underlie FF fluctuations.

As shown in Figure 3d, PCE progressively increases as defect density in the absorber layer decreases. The peak PCE of 17.91% occurs at a defect density of 10^13^ cm^−3^, accompanied by an FF of 78.59%. CCSC defect density remains relatively low [42], though specific numerical characterization reports remain scarce. Conversely, for CTI, there are constructive insights that emerge from defect engineering perspectives. Urmi et al.’s density functional theory (DFT) analysis of 12 potential point defects underscores the necessity to rigorously suppress the total defect density below 10^14^ cm^−3^, while avoiding I-poor/Ti-rich conditions to mitigate the formation of deleterious deep-level defects such as Ti interstitials. Under I-rich/Ti-poor synthesis, process refinement is critical to minimize I vacancy formation [20]. In alignment with these findings and supported by our simulation data, this work recommends maintaining defect densities of less than 10^14^ cm^−3^ for practical fabrication. Nevertheless, our simulations prioritize establishing an efficiency value that closely approaches the theoretical maximum achievable in experimental contexts. Thus, the 10^13^ cm^−3^ density is ultimately selected for optimal property calculation. For future PSC designers, they should meticulously regulate defect density and optimize heterojunction bands collaboratively.

### 3.3. Impact of Absorber Layer Thickness on Properties

The thicknesses of the CTI and CCSC layers directly impact the entire cycle of carrier generation, transport, and collection, characterized by the following competing mechanisms. Increasing the CTI thickness enhances the capture efficiency of high-energy photons. However, when it exceeds the carrier diffusion length, bulk recombination intensifies, inhibiting Fermi-level splitting and thereby decreasing V_oc_ [43]. Similarly, the CCSC layer thickness must be optimized to leverage its narrow-gap properties for improved low-energy photon absorption. Nevertheless, excessive thickness weakens the intrinsic electric field at the heterojunction boundary, thereby hindering the separation of photogenerated carriers [44]. Furthermore, the thickness ratio of the dual-absorber layer regulates the heterojunction’s energy band gradient, impacting directional carrier transport. Where a stepped band arrangement forms at the CTI/CCSC interface, a self-driven carrier transport channel is established, enhancing J_sc_. Conversely, band alignment disruption arising from thickness imbalance contributes to interfacial carrier accumulation, exacerbating non-radiative recombination and reducing V_oc_ [45].

**Figure 4:**
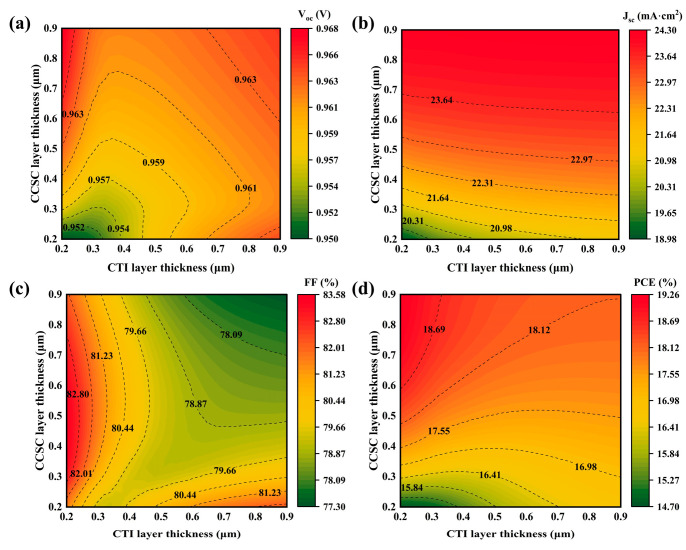
Impact of absorber layer thickness on PSC properties: (**a**–**d**) influence on V_oc_, J_sc_, FF, and PCE.

Figure 4a–d depict the impact of the absorber layer thickness ranging from 0.2 μm to 0.9 μm on the properties of PSC. The erratic changes in FF can be attributed to the competing factors described above. By contrast, the PCE variation is more predictable, reaching a maximum value of 19.25% at a thickness of 0.2 μm for CTI and 0.9 μm for CCSC, respectively, with an FF of 82.13%. Considering the diffusion lengths of both CCSC and CTI [46,47], along with the optimized thicknesses falling within these characteristic lengths, efficient charge extraction is physically guaranteed [48]. This correlation provides strong justification for the rationality of the simulated outcomes. Thus, subsequent PSC design should account for both absorber layer thickness and energy band engineering.

### 3.4. Impact of Interface Defect Density of Absorber Layer on Properties

The properties of PSC are significantly impacted by defect density at the CTI/CCSC interface through band structure and carrier transport mechanisms. High defect density at the heterojunction interface induces localized band bending, thereby disrupting the band gradient. This, in turn, accelerates non-radiative recombination of photo-generated carriers at the interface, ultimately diminishing V_oc_. Simultaneously, interface defects, functioning as scattering centers, impede carrier movement across the interface, leading to carrier accumulation within the absorber layer, which exacerbates FF attenuation. Furthermore, the space charge effect triggered by defects can degrade the selective carrier extraction ability of ETL and HTL, diminishing separation efficiency for both electrons and holes [49] and consequently constraining J_sc_.

**Figure 5:**
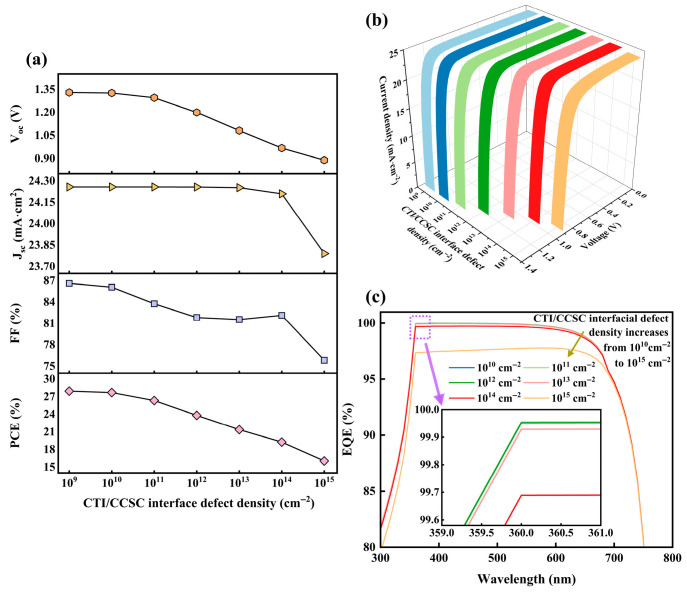
Impact of absorber layer interface defect density on PSC: (**a**) impact on PSC properties; (**b**) impact on J–V curve; (**c**) impact on the EQE curve.

Figure 5a illustrates the impact on PSC property parameters as interface defect density ranges from 10^9^ cm^−2^ to 10^15^ cm^−2^. While FF generally declines with increasing defect density, it increases at 10^14^ cm^−2^, a phenomenon ascribed to the competitive regulatory mechanism of defects at the CTI/CCSC interface. Upon defect density reaching a specific threshold, a defect-induced quantum dot-like tunneling network forms at the interface, enhancing hole tunneling probability. This partially offsets the carrier lifetime loss caused by defect recombination. However, this phenomenon is transient. At higher defect density, recombination centers increase substantially, completely compromising carrier separation efficiency [50]. Moreover, PCE decreases progressively with rising interface defect density, achieving its maximum value of 27.94% at 10^9^ cm^−2^, accompanied by an FF of 86.61%. Given the achievable 10^13^ cm^−3^ bulk defect density, attaining this interface defect density value appears promising [51].

Figure 5b,c depict changes in current–voltage (J–V) curves and external quantum efficiency (EQE) with defect density. External quantum efficiency, quantifying the proportion of incident photons converted into external circuit current, encompasses light absorption capability, carrier generation, and collection. As defect density increases, both of them exhibit a negative trend. Consequently, PSC designers should prioritize regulating interface defect density.

### 3.5. Impact of Band Gap of Absorber Layer on Properties

The absorber layer band gap impacts PSC properties through spectral coverage and carrier transport mechanisms. Regarding individual contributions, the wide band gap of CTI creates an elevated intrinsic potential at the boundary, which elevates V_oc_. Conversely, the narrow band gap of CCSC extends long-wave photon absorption, facilitating photo-generated carrier production [52] and thereby increasing J_sc_. Furthermore, the disparity in band gap between the dual-absorber layers enhances photo-generated carrier separation via a cascade band arrangement. This phenomenon reduces the interface barrier while suppressing carrier accumulation and recombination, ultimately improving FF [14]. Finally, the band alignment of HTL and ETL requires coordination to prevent carrier reverse diffusion.

**Figure 6:**
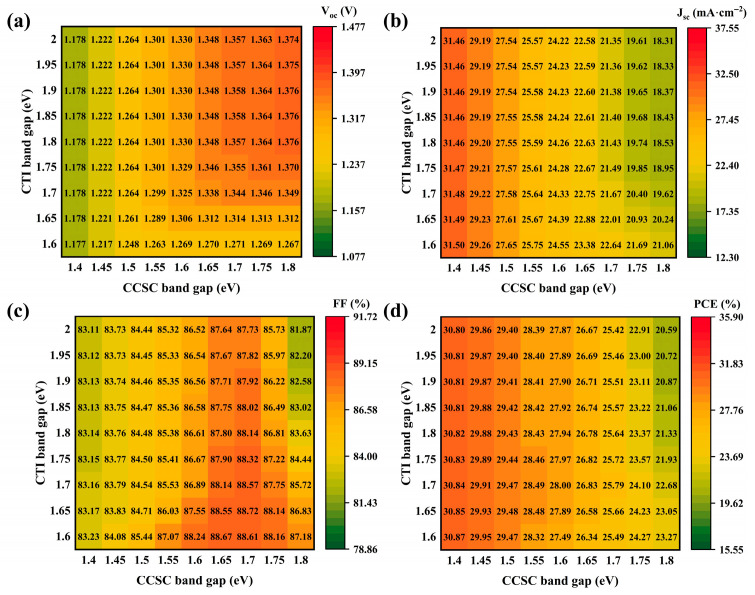
Impact of absorber layer band gap on PSC properties: (**a**–**d**) influence on V_oc_, J_sc_, FF, and PCE.

Figure 6a–d depict variations in PSC property parameters, with the CTI band gap ranging between 1.6 eV and 2 eV [53] and the CCSC band gap between 1.4 eV and 1.8 eV [54,55]. As the CTI band gap increases, FF demonstrates a gradual upward trend. However, with increasing CCSC band gap, FF initially rises and then declines, a behavior attributable to carrier transmission and recombination mechanisms. When the CCSC band gap is narrow, the energy-level gradient between CCSC and CTI enhances FF. Nevertheless, as the CCSC band gap further widens, this energy-level gradient diminishes, reducing the driving force for carrier separation and causing FF deterioration. Concurrently, the expansion of the CCSC band gap increases energy-level mismatch with HTL, creating an interface barrier that elevates carrier reverse recombination probability, further reinforcing FF attenuation [56]. Concerning PCE, it is primarily modulated by the CCSC band gap. The optimal PCE of 30.87% occurs at band gaps of 1.4 eV for CCSC and 1.6 eV for CTI, accompanied by an FF of 83.23%. According to the literature records, the optimized band gap values can be achieved [57,58]. Consequently, future PSC designers should emphasize a precise balance between energy band gradients and interface dynamics.

### 3.6. Impact of Back Electrode on Properties

PSC properties are impacted by the back electrode work function, which dictates the matching degree at the HTL/electrode interface. Where a favorable ohmic contact forms, the hole extraction barrier can be significantly reduced, thereby diminishing non-radiative recombination loss [59]. Conversely, mismatched work functions induce Schottky barrier formation at the interface, leading to hole accumulation near the HTL/electrode junction. This accumulation exacerbates carrier recombination and constrains the built-in electric field distribution [60].

**Figure 7:**
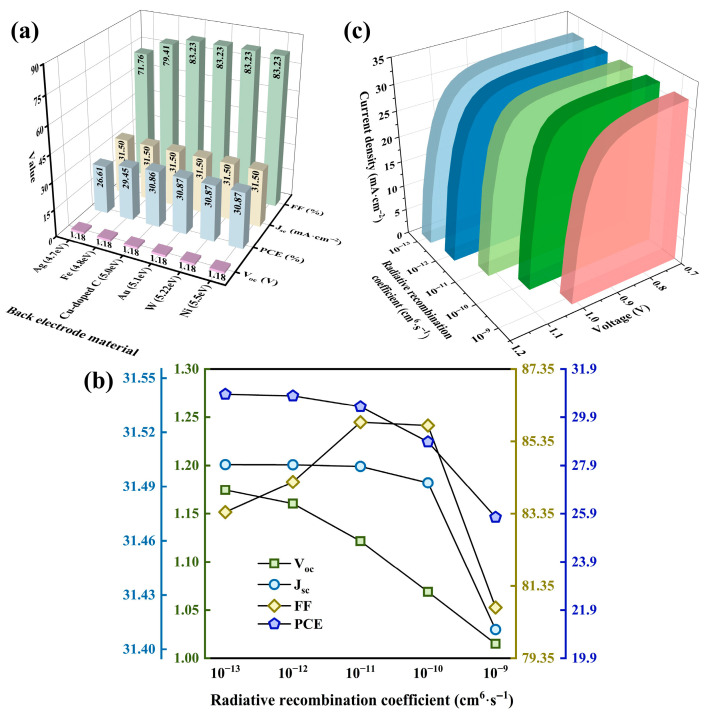
(**a**) Impact of back electrode on property parameters of PSC; (**b**) impact of radiation recombination coefficient on property parameters of PSC; (**c**) impact of radiation recombination coefficient on J–V curve of PSC.

Figure 7a depicts variations in property parameters as the work function ranges from 4.7 eV to 5.5 eV [7,61]. Considering material cost and stability, Cu-doped C is selected as the back contact material, achieving a PCE of 30.86% and an FF of 83.23%.

### 3.7. Impact of Radiative Recombination on Properties

Radiation recombination, as the dominant recombination mechanism in ideal PSCs, significantly impacts device property parameters. A lower radiative recombination rate enhances the quasi-Fermi level splitting, increasing V_oc_. However, its direct impact on J_sc_ remains limited. In this section, the radiation recombination coefficient (*B*) is simulated across 10^−13^ cm^6^·s^−1^ to 10^−9^ cm^6^·s^−1^, as depicted in Figure 7b. Notably, FF initially elevates but subsequently diminishes with rising *B*. This trend arises because, at lower *B* values, radiative recombination generally becomes the governing mechanism. Non-radiative recombination is thereby suppressed, extending carrier lifetime, reducing series resistance, and improving carrier extraction efficiency—collectively enhancing FF. This also explains the marginally higher PCE observed at low *B* compared to the radiation-free scenario. Conversely, high *B* values significantly accelerate radiation recombination, promoting carrier loss and diminishing charge collection efficiency, which lowers FF. Furthermore, Figure 7c illustrates the J–V curve evolution with *B*, clearly demonstrating reduced current density at identical voltages for larger *B* [62].

In subsequent simulations, *B* is ultimately represented by Equation (4) for each material. In the equation, *N_c_* represents the maximum number of quantum states that can accommodate electrons in the conduction band, *N_v_* indicates the number of states in the valence band that can be occupied by holes, *K_B_* signifies a thermodynamic constant, *T* denotes the absolute temperature, and *d* refers to the physical thickness of the active layer. After adopting the aforementioned conditions, the PCE decreased to 30.52%, with a corresponding FF of 85.84%. Therefore, future PSC designers should pay attention to the material properties linked to radiative recombination.

(4) $$B=\frac{2\pi c}{N_{V}N_{C}}\left(\frac{1}{d}\right){\left(\frac{E_{g}}{k_{B}T}\right)}^{2}{\left(\frac{k_{B}T}{hc}\right)}^{3}$$

### 3.8. Impact of Resistance on Properties

Series resistance (R_s_) and shunt resistance (R_sh_) are inherent in PSCs. R_s_ primarily arises from interfacial carrier transport losses, encompassing obstruction at electrode and heterojunction interfaces, alongside bulk transport losses such as the resistance within the FTO material. Conversely, R_sh_ is dominated by leakage currents originating from bulk phase defects in the active layer and recombination channels at grain boundaries or interlayer interfaces [63].

While R_s_ impedes the directional migration of photogenerated carriers, contributing to ohmic losses that degrade FF, its impact on V_oc_ is relatively minor, as V_oc_ is predominantly governed by Fermi-level splitting. Under standard illumination, the ohmic drop associated with R_s_ is typically small, allowing carrier transport interference to be neglected [64]. Consequently, J_sc_ exhibits less sensitivity to R_s_. In contrast, R_sh_ induces direct lateral carrier leakage and recombination losses, leading to significant attenuation in property parameters [65].

**Figure 8:**
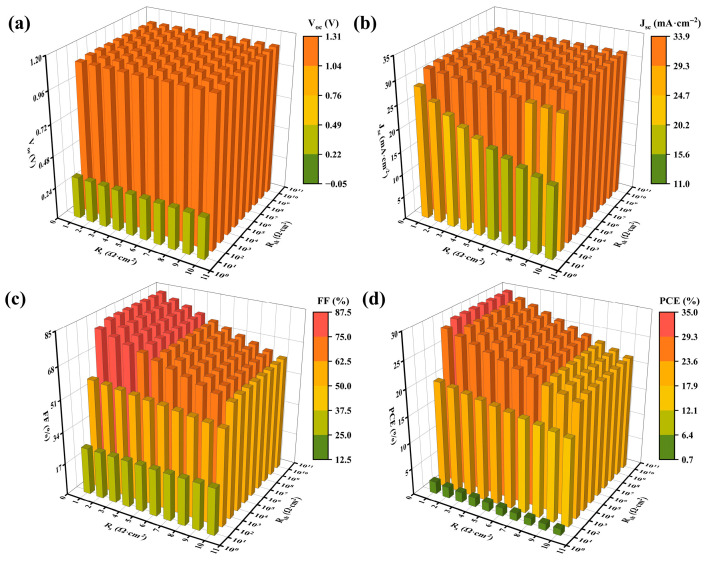
Impact of R_s_ and R_sh_ on PSC properties: (**a**–**d**) impact on V_oc_, J_sc_, FF, and PCE.

As revealed in Figure 8, R_s_ is simulated within 1–10 Ω·cm^2^ and R_sh_ within 10^1^–10^10^ Ω·cm^2^. Overall, PCE reduction due to R_s_ remains generally controllable, whereas extremely low R_sh_ (10^1^ Ω·cm^2^) contributes to catastrophic device efficiency decline. Fortunately, the practical device R_s_ approximates 3 Ω·cm^2^ and R_sh_ about 10^4^ Ω·cm^2^ [66]. Under these conditions, the PCE attains 27.65%, with an FF of 77.89%. Therefore, future PSC design should prioritize optimizing interface band arrangements while mitigating bulk and interface recombination.

### 3.9. Impact of Light Intensity on Properties

Under real-world conditions, solar irradiance fluctuates significantly due to seasonal and geographical factors. Consequently, examining the device’s response to light intensity allows evaluation of its stability and durability. The impact of light intensity on device properties primarily involves carrier dynamics and interface behavior. With increasing light intensity, the concentration of photo-generated carriers within the absorber layer rises substantially, enhancing the quasi-Fermi-level splitting and thus V_oc_ [67]. The photogenerated carrier density increases with light intensity, contributing to a nearly linear rise in J_sc_. Furthermore, the rising photogenerated current causes power loss attributed to R_s_ to increase nonlinearly, inducing a significant ohmic voltage drop near the maximum power point, which consequently reduces FF [63].

**Figure 9:**
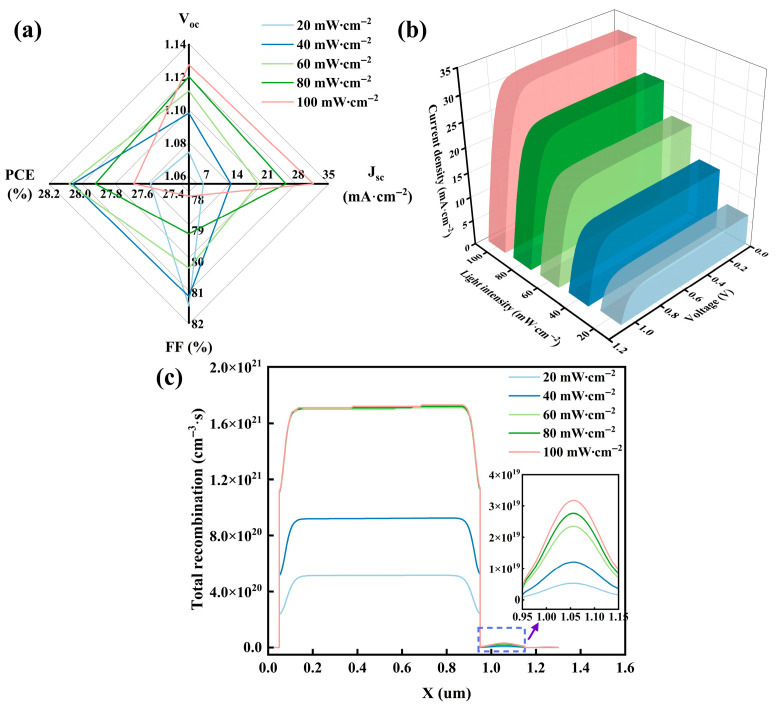
Impact of light intensity and back electrode on PSC: (**a**) impact of light intensity on PSC property parameters; (**b**) impact of light intensity on J–V curve; (**c**) impact of light intensity on recombination rate.

Figure 9a–c illustrate the variation in PSC property parameters, recombination rate, and J–V curves as light intensity increases from 20 to 100 mW·cm^−2^. From the figure, the nonlinear PCE alteration stems from divergent responses in J_sc_ and FF, while the recombination rate initially rises before stabilizing with increasing light intensity, with the J–V curve progressively improving. The recombination mechanisms encompass radiative recombination and non-radiative recombination, with the latter primarily governed by SRH recombination as described by Equations (5) and (6), where $n_{e,h}$ represents the local density of electrons in the conduction band and holes in the valence band, $n_{i}$ symbolizes the natural concentration of electron–hole pairs in thermal equilibrium, $\tau_{e,h}$ stands for the free motion time of carriers before being captured, $v_{th}$ denotes the disorderly motion rate of carriers under thermal disturbance, $\sigma_{e,h}$ indicates the effective capture cross-section of electrons and holes by defects, and $N_{defeat}$ signifies the density of defect states able to trap carriers in the material.

The enhancement in recombination rate stems from the considerable elevation in the quantity of photo-generated carriers when the light intensity enlarges. Nevertheless, as the light intensity fluctuates within the range of 60 mW·cm^−2^ and 100 mW·cm^−2^, the recombination rate remains nearly constant, driven by the dynamic equilibrium of the carrier and the saturation mechanism of the defect state. As the light intensity grows within this range, the band gradient at the interface of the dual-absorber layer and the properties of the charge transport layer continuously optimize the photo-generated carrier separation efficiency along with carrier transport efficiency. More critically, under high injection conditions, the defect states in the absorber layer are progressively filled, bringing about the defect-assisted recombination to approach saturation [62]. Hence, the dependence of the recombination rate on light intensity lessened.

It is noteworthy that the impact of light intensity on PCE is approximately 1.5%, which showcases the excellent light adaptability of the PSC. Since the essence of FTO reflection is similar to the mechanism of light intensity, a 4% FTO reflection is set to better approximate real-world conditions [68]. Under this configuration, the device reaches a PCE of 26.60% and an FF of 78.15%. Accordingly, subsequent PSC designers ought to be mindful of carrier homeostasis regulation to obtain better properties under different light intensities.

(5) $$R_{SRH}=\frac{n_{e}n_{h}-n_{i}^{2}}{\tau_{e}\left(\frac{n_{e}+n_{i}}{\tau_{h}}+\frac{n_{h}+n_{i}}{\tau_{e}}\right)}$$

(6) $$\tau_{e,h}=\frac{1}{v_{th}\cdot \sigma_{e,h}\cdot N_{\mathrm{defect}}}$$

### 3.10. Impact of Temperature on Properties

In practical outdoor scenarios, PSCs are subjected to severe temperature fluctuations, such as the day–night cycle. Consequently, this section concentrates on the impact of temperature fluctuations on PSC. With the rise in temperature, the lattice thermal vibration in the absorber layer intensifies, which, in turn, elevates the non-radiative recombination probability among photo-generated carriers. Subsequently, the resulting band disorder, along with the suppression of Fermi-level splitting caused by the increase in defect density, is observable as the decline in V_oc_ [69]. Meanwhile, driven by the varying thermal expansion coefficients of CTI and CCSC, the additional stress introduced at the interface hinders the carrier transport efficiency. Nevertheless, temperature rise contributes to the reduced localized effect of carriers and the increased dissociation efficiency of photo-generated carriers. Owing to these two factors combined, J_sc_ exhibits a minor variation range [70]. Moreover, at elevated temperatures, the anisotropy of carrier mobility intensifies, making for obstruction in the transmission path. Simultaneously, a high interfacial defect-assisted recombination rate destroys the carrier concentration gradient and the synchronization of charge extraction [71], which is evident in the decrease in FF.

**Figure 10:**
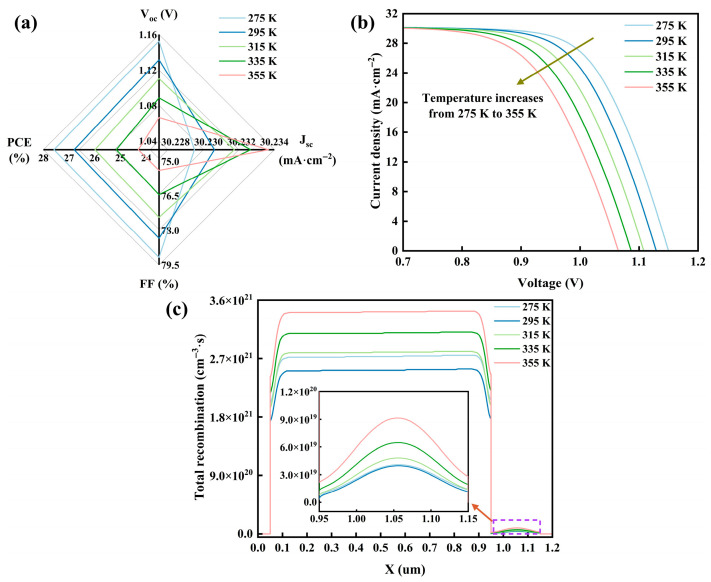
Impact of temperature on PSC: (**a**) impact on PSC property parameters; (**b**) impact on J–V curves; (**c**) impact on recombination rate.

Figure 10a–c depict the variation of PSC property parameters, the J–V curve, and the recombination rate with temperature at 275 K–355 K. As temperature rises, FF and PCE progressively decrease, with the current under the same voltage also gradually diminishing. However, the recombination rate does not vary linearly with the temperature.

The nonlinear variation of the recombination rate with temperature can be ascribed to the temperature-dependent recombination dynamics in the absorber layer. In the CTI layer, at low temperature, the contraction of the [TiI_6_]^2−^ octahedral structure promotes the migration of iodine vacancies along the specific lattice directions, with the resulting defect channels expediting the recombination of carriers [6]. Conversely, at high temperature, structure tension makes for vacancy recombination and partial annihilation, thereby diminishing the defect-assisted recombination effect. In the CCSC layer, the distribution of Cl vacancies in the [CuSb_2_Cl_12_]^4−^ unit can be modulated by temperature variation, which, in turn, gives rise to the carrier trap shift dynamically between deep and shallow energy levels [72]. Consequently, the rate of carrier recombination in the dual-absorber layer exhibits fluctuations.

Notably, the reduction in PCE facilitated by raised temperature is maintained at around 3%, while the alteration in PCE at low temperature is controlled within 8% (relative to 300 K), highlighting the excellent stability of PSC. Overall, the thermal expansion coefficient of materials and the dynamic management of defects should be taken into consideration by subsequent PSC designers in order to achieve better PCE.

### 3.11. Multiple Algorithm-Driven PSC Property Research

ML includes 1000 initial data points, as indicated in Table 2, with 800 allocated to training and 200 to testing. Algorithm comparisons include LR, RF, and XGBoost.

**Figure 11:**
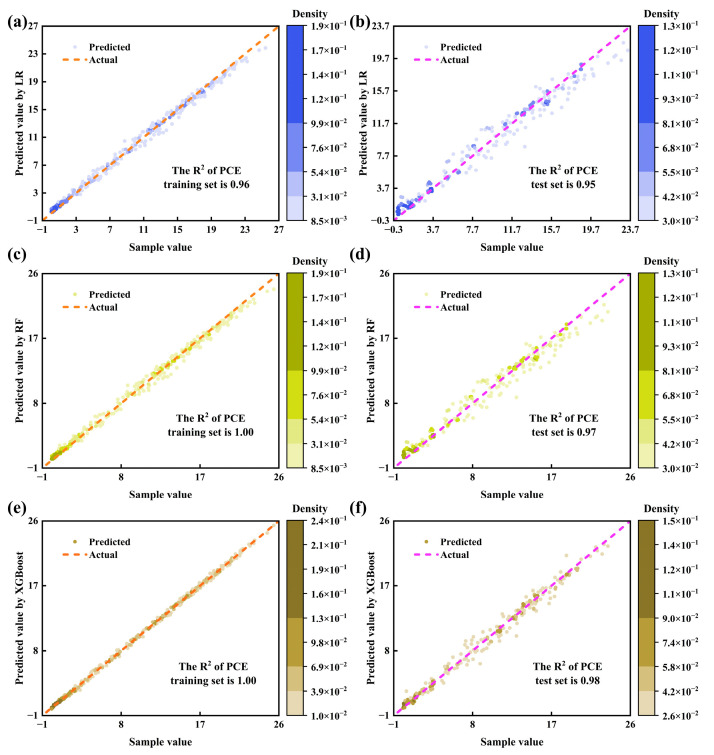
Scatter plots comparing predicted/actual values of PCE for training/test data: (**a**,**b**) predicted by LR; (**c**,**d**) predicted by RF; (**e**,**f**) predicted by XGBoost.

For LR, two scenarios are evaluated: one incorporating several nonlinear terms and another utilizing solely linear terms. The former employs 42 independent variables, comprising 7 original variables, 7 quadratic terms, 7 cubic terms, and 21 interaction terms. Crucial prediction metrics are R^2^ and RMSE. R^2^ quantifies a model’s capacity to explain data variance, reflecting relative predictive accuracy. Furthermore, R^2^ approaching 1 indicates superior properties, with values over 0.85 signifying good model fit. RMSE measures absolute error between predicted and actual values, where values nearer to 0 are preferable. As Table 3 demonstrates, adopting nonlinear terms substantially enhanced prediction performance, particularly in FF. Surprisingly, it outperformed RF in the prediction of V_oc_. However, existing terms cannot fully predict FF variations, stemming from characteristics associated with bulk defects’ dual nature and the bandgap threshold regulation mechanism. Improvement is anticipated by introducing additional nonlinear terms, such as exponential and logarithmic terms. Subsequently, Figure 11a,b presents PCE fitting using nonlinear terms, revealing overall minor errors. Fitting results for the remaining indicators appear in Supplementary Information Section SD.

Regarding tree ensemble algorithms, including RF and XGBoost, relevant parameter settings are provided in Table 4, with prediction properties summarized in Table 3. RF outperforms LR on most metrics. Conversely, XGBoost, leveraging intrinsic residual learning and regularization, further improved upon RF. For FF prediction, XGBoost effectively captured the correct nonlinear relationship, achieving a test-set R^2^ of 0.92. This indicates the model now explains most FF variations, addressing LR’s limitation. Figure 11c–f illustrates the fitting of RF and XGBoost for PCE, with results for other variables in Supporting Information Section SD.

**Figure 12:**
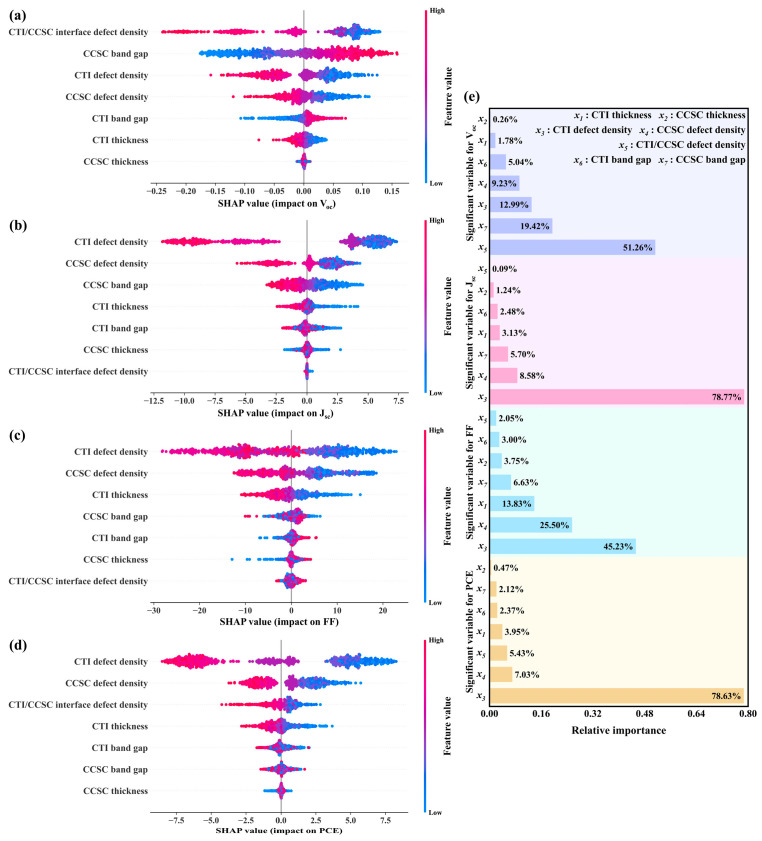
(**a**–**d**) SHAP graphs for each property parameter; (**e**) the contribution of each variable for each property parameter.

Subsequently, the SHAP algorithm analyzed the XGBoost model, as revealed in Figure 12. Results indicate that for V_oc_, the absorber layer interface defect density exerts the greatest influence, with an importance of approximately 51.26%. For J_sc_, FF, and PCE, CTI layer defect density is most impactful, with impact levels of 78.77%, 45.23%, and 78.63%, respectively. Thus, future research developing similar-structure PSCs should prioritize investigating absorber layer interface parameters identified as highly influential.

### 3.12. Brief Discussion of Actual Factors

Due to SCAPS-1D limitations, conducting a quantitative analysis of factors in Section 2.1 proves infeasible, permitting only a brief discussion herein.

As illuminated in Figure 9c, recombination predominantly occurs within the absorber layer, primarily comprising radiative recombination, SRH recombination, and Auger recombination. However, as He et al. demonstrate, Auger recombination remains negligible for materials with doping concentrations below 10^17^ cm^−3^ [62]. Consequently, it is excluded from this study.

**Figure 13:**
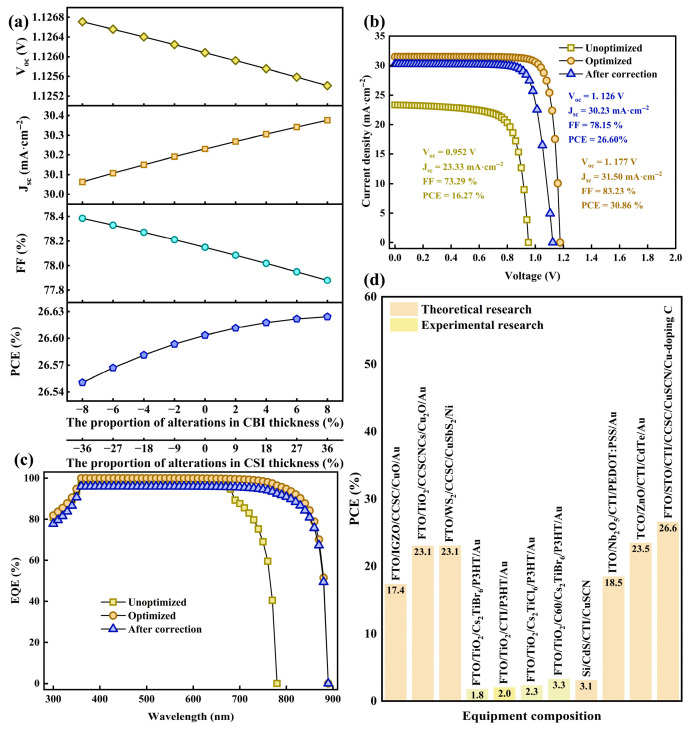
(**a**) The impact of uneven thickness on performance parameters; (**b**) comparison of J–V curves before optimization, after optimization, and following correction; (**c**) comparison of EQE curves before optimization, after optimization, and following correction; (**d**) comparisons with other studies.

Regarding lateral non-uniformities, analysis focuses primarily on thickness variation. Limited by SCAPS-1D, only simplified simulations were performed. Given typical thickness non-uniformity approximating 8% [73], this work simulates scenarios where the total absorber layer thickness remained constant while the CCSC thickness varied by ±8%, as illuminated in Figure 13a. These results indicate that PCE does not change significantly within this range.

In interface roughness studies, Zuñiga et al. characterized transmission and reflection haze effects at layer interfaces through Silvaco modeling [74]. Analogous to dual-absorber layer structures, interfacial roughness significantly impacts device performance—most notably at the dual-absorber layer interface, followed by HTL/absorber layers, whereas ETL/absorber layers exhibit lower sensitivity. While roughness may exacerbate interface recombination, controlled roughness enhances EQE via light reflection. For dual-absorber layers, maintaining the root mean square of thickness below 80 nm is recommended. Current data indicate CCSCNCs’ surface roughness ranges between 2–8 nm [42,75], whereas CTI-related records remain limited. Thus, experimental designs are suggested to precisely control interfacial roughness within this optimized range, balancing light-trapping and recombination suppression.

Temperature stability undergoes quantitative assessment in Section 3.10. Concerning humidity stability, CCSC exhibits strong resilience [76], whereas CTI requires avoidance of humid environments [77].

For fabrication, both the charge transport layer and the absorber layer can be fabricated via spin-coating, followed by annealing [42,78,79,80]. Furthermore, extensive experimental work exists regarding dual-absorber layer manufacturing [81,82,83], yielding numerous suggestions. Zhang et al.’s research highlights the significance of interface engineering, which significantly diminishes recombination losses. Additionally, band alignment proves equally critical for structures [84].

Nevertheless, incorporating all other practical factors remains unfeasible. Thus, this study serves primarily as an experimental reference, with the simulated PCE representing an almost upper-limit benchmark.

### 3.13. Optimized Device Properties

The efficiency of the optimized device attains 26.60%, with V_oc_ reaching 1.13 V, J_sc_ being 30.23 mA·cm^−2^, and FF amounting to 78.15%. Figure 13b,c present a comparison between the J–V curve and the EQE curve before optimization, after optimization, and following correction, demonstrating a notable enhancement subsequent to the optimization process. The EQE improvement was predominantly observed in the near-infrared segment, ranging from 750 nm to 850 nm, resulting from the reduced band gap. Meanwhile, the transverse comparison in Figure 13d reveals that, in contrast with other studies based on CCSC bulk materials, CCSCNCs, and Cs_2_TiX_6_ (X = Cl, Br, I), this work exhibits substantial efficiency improvements, with more precise information presented in Section SE of Supporting Information.

## 4. Conclusions

This work employed the SCAPS-1D simulation platform for investigation. Given that the SQ limit substantially constrains the maximum efficiency attainable by single-absorber layer PSCs, this research adopts a dual-absorber layer configuration, designing a Glass/FTO/ETL/CTI/CCSC/HTL/Au architecture. Following analysis of band alignment, PCE, and interfacial compatibility, STO and CuSCN were selected from 26 charge transport layer candidates. Subsequent examination encompassed band diagrams alongside absorption spectra, yielding an initial PCE of 16.23%. Subsequently, optimization was performed on absorber layer thickness, bulk defect density, bandgap, and interface defect density. Concurrently, the back electrode was substituted with Cu-doped carbon, considering cost, efficiency, and stability considerations, achieving a notable PCE of 30.86%. During optimization, detailed recurrent analysis elucidated the FF nonlinear variation. Further assessment of radiative recombination, resistance, and FTO reflectance adjusted the PCE to 26.60%, corresponding to a V_oc_ of 1.13 V, J_sc_ of 30.23 mA/cm^2^, and FF of 78.15%. Device performance evaluation across the 275 K–355 K temperature range demonstrated exceptional thermal stability. Following the evaluation, three machine learning algorithms—LR, RF, and XGBoost—were comparatively employed for predicting the property parameters of PSCs. XGBoost exhibited superior performance, with R^2^ exceeding 0.99 in most models. In addition, SHAP analysis identified the defect density of CTI as the predominant efficiency determinant, providing optimization guidance for structurally analogous devices. Finally, qualitative analysis addressed non-ideal factors excluded from efficiency calculations due to software constraints. Furthermore, all incorporated materials can be deposited via mature processes, reducing experimental complexity. Substantial theoretical foundations exist for these materials, enhancing experimental reproducibility and result verifiability while ensuring the proposed structure’s high-efficiency reliability, thereby advancing perovskite research.

## Figures and Tables

**Table 1:** Lattice matching of HTL and absorber layer.

Material	a (Å)	b (Å)	c (Å)	***δ*** (%)
CCSC	13.083	7.3507	13.070	–
NiO	4.174	4.174	4.174	27.56
CuSCN	3.85	–	10.938	8.88

**Table 2:** The initial data for ML.

Type	Parameters	Data Number				
		1	2	3	…	1000
Independent variable	CTI thickness (μm)	0.2	0.7	0.4	…	0.8
	CCSC thickness (μm)	0.3	0.5	0.2	…	0.4
	CTI defect density (cm^−3^)	1 × 10^16^	1 × 10^14^	1 × 10^18^	…	1 × 10^18^
	CCSC defect density (cm^−3^)	1 × 10^17^	1 × 10^17^	1 × 10^13^	…	1 × 10^18^
	CTI/CCSC interface defect density (cm^−3^)	1 × 10^12^	1 × 10^13^	1 × 10^14^	…	1 × 10^11^
	CTI band gap (eV)	1.6	1.84	1.89	…	1.67
	CCSC band gap (eV)	1.44	1.67	1.62	…	1.54
Dependent variable	V_oc_ (V)	0.9716	1.1282	0.9061	…	0.9044
	J_sc_ (mA·cm^−2^)	23.0985	18.8206	5.9881	…	0.8438
	FF (%)	56.7202	69.6902	69.7301	…	31.6132
	PCE (%)	12.7296	14.7969	3.7833	…	0.2413

**Table 3:** Property indicators of each algorithm.

Regression Type	Data Type	Evaluation Type	Property Parameter			
			V_oc_ (V)	$J_{\mathrm{sc}}\;(\mathrm{mA}\cdot$cm^−2^)	FF (%)	PCE (%)
LR (Excluding nonlinear terms)	Training set	R^2^	0.8097	0.7771	0.4220	0.8148
		RMSE	0.0696	3.6341	13.2479	2.7795
	Test set	R^2^	0.7727	0.8334	0.3483	0.8210
		RMSE	0.0770	3.2136	13.9988	2.6867
LR (including nonlinear terms)	Training set	R^2^	0.9625	0.9353	0.7233	0.9575
		RMSE	0.0309	1.9575	9.1671	1.3312
	Test set	R^2^	0.9422	0.9447	0.6961	0.9460
		RMSE	0.0388	1.8519	9.5591	1.4755
RF	Training set	R^2^	0.9912	0.9958	0.9831	0.9954
		RMSE	0.0149	0.5011	2.2629	0.4367
	Test set	R^2^	0.9308	0.9812	0.8886	0.9671
		RMSE	0.0425	1.0784	5.7889	1.1521
XGBoost	Training set	R^2^	0.9980	0.9991	0.9921	0.9993
		RMSE	0.0071	0.2299	1.5440	0.1742
	Test set	R^2^	0.9771	0.9904	0.9188	0.9845
		RMSE	0.0244	0.7725	4.9410	0.7908

**Table 4:** The parameter settings adopted by RF and XGBoost.

Regression Type	Parameter	Value	Function
RF	Decision tree number	300	Determine the accuracy and robustness of the model
	The minimum sample size of leaf nodes	1	Control the complexity of a single tree
XGBoost	Decision tree number	300	Determine the complexity of the model and the time consumed for training
	Learning rate	0.05	The contribution weight of each tree to the prediction result
	Maximum depth of the decision tree	5	Limit the number of branch levels of the tree to control the complexity of feature interaction
	Random number seed	42	Fix all random processes, including feature and data sampling

## Data Availability

Data will be made available on request.

## Supplementary Materials

The following supporting information can be downloaded at: https://www.mdpi.com/article/10.3390/nano15161245/s1, Table S1. Initial data for reproduction. Table S2. Input parameters of the absorber layer and FTO. Table S3. Input parameters of interface. Table S4. Input parameters of ETL. Table S5. Input parameters of HTL. Figure S1. The fitting situation of LR for V_oc_, J_sc_ and FF. Figure S2. The fitting situation of RF for V_oc_, J_sc_ and FF. Figure S3. The fitting situation of XGBoost for V_oc_, J_sc_ and FF. Table S6. Comparisons with other studies. Refs. [85,86,87,88,89,90,91,92,93,94,95,96,97,98,99,100,101,102,103,104,105,106,107,108,109,110,111,112,113] are cited in the supplementary materials.
