# Supplementary material for: Optimizing Inorganic Cs4CuSb2Cl12/Cs2TiI6 Dual-Absorber Solar Cells: SCAPS-1D Simulations and Machine Learning
Source: Nanomaterials (Basel). 2025 Aug 14;15(16):1245. doi: 10.3390/nano15161245 (PMC12388759; doi:10.3390/nano15161245)
Supplement: Supplementary file 1 [file nanomaterials-15-01245-s001.zip › nanomaterials-3750533-supplementary.pdf]

*Supporting Information*

# Optimizing Inorganic Cs4CuSb2Cl12/Cs2TiI6 Dual-Absorber Solar Cells: SCAPS-1D Simulations and Machine Learning

*Xiangde Li[1], Yuming Fang[1], Jiang Zhao[1,2,*]*

[1]College of Integrated Circuit Science and Engineering, Nanjing University of Posts and Telecommunications, Nanjing 210023, China

[2]Nantong Institute, Nanjing University of Posts and Telecommunications, Nantong 226006, China

*Corresponding author, E-mail: jzhao@njupt.edu.cn (J. Zhao)

## SA. SCAPS-1D optimization

SCAPS-1D software is based on the drift-diffusion model, and is solved according to Poisson equation (Eq. S1), electron current density expression (Eq. S2), hole current density expression (Eq. S3), electron continuity equation (Eq. S4), hole continuity equation (Eq. S5). The steady state and transient electrical responses of perovskite solar cells (PSCs) under standard test conditions (AM1.5G spectrum, irradiance 1000 W/m², temperature 300 K) can be obtained by solving the partial differential equation simultaneously. The photoelectric conversion efficiency (PCE) is expressed in Eq. S6.

$$\frac{d^2\psi(x)}{dx^2} = -\frac{q}{\varepsilon_0\varepsilon_r}\left[-n(x)+p(x)+N_D^+(x)-N_A^-(x)+\frac{\rho_{def}}{q}\right] \quad (S1)$$

$$J_n = n\mu_n\frac{d\psi}{dx}+D_n\frac{\partial n}{\partial x} \quad (S2)$$

$$J_p = p\mu_p\frac{d\psi}{dx}+D_p\frac{\partial p}{\partial x} \quad (S3)$$

$$-\frac{\partial J_n}{\partial x}-R_n+G=\frac{\partial n}{\partial t} \quad (S4)$$

$$-\frac{\partial J_p}{\partial x}-R_p+G=\frac{\partial p}{\partial t} \quad (S5)$$

$$\mathrm{PCE}=\frac{P_{\mathrm{max}}}{P_{\mathrm{in}}}=\frac{J_{sc}\times V_{oc}\times \mathrm{FF}}{P_{\mathrm{in}}}\times 100\% \quad (S6)$$

In the above equation, $\psi$ represents the electrostatic potential, $\varepsilon_0$ represents the dielectric constant of the vacuum, and $\varepsilon_r$ represents the relative dielectric constant of the semiconductor. $q$ represents the electron charge, where $p$ represents the density of holes and $n$ represents the equilibrium concentration of holes and electrons. $N_D^+$ refers to the equilibrium concentration of the ionizing donor, while $N_A^-$ refers to the equilibrium concentration of the ionizing acceptor. The charge density is $\rho$, $J_n$ represents the current density of the electron, and $J_p$ represents the current density of the hole. $R_n$ and $R_p$ are the compound rate of electrons and holes, while $G$ denotes the formation rate. $x$ denotes the position along the longitudinal axis of the device, and $t$ denotes the time. In addition, $D_n$ and $D_p$ are the diffusivity of carriers, while $\mu_n$ and $\mu_p$ represent the mobility of electrons and holes.

## SB. Initial data for reproduction

The experiment described in the original paper was reproduced using SCAPS-1D simulations with the following parameter set: a series resistance value of 8.6 $\Omega \cdot cm^2$, a shunt resistance of 1200 $\Omega \cdot cm^2$, and a front-contact (FTO) reflection coefficient of 4%, with input parameters of each layer illuminated in Table S1.

Table S1. Initial data for reproduction.

| Parameters | Spiro-O MeTAD | IDL2 | $CH_3NH_3PbI_3$ | IDL1 | $TiO_2$ | FTO |
|---|---|---|---|---|---|---|
| Thickness(μm) | 0.45 | 0.005 | 0.65 | 0.005 | 0.04 | 0.65 |
| $E_g$(eV) | 3 | 1.5 | 1.5 | 1.5 | 3.26 | 3.5 |
| χ(eV) | 2.45 | 3.9 | 3.9 | 3.9 | 4.2 | 4 |
| $\varepsilon_r$ | 3 | 10 | 10 | 10 | 10 | 9 |
| $N_c(cm^{-3})$ | $2.2\times10^{18}$ | $2.75\times10^{18}$ | $2.75\times10^{18}$ | $2.75\times10^{18}$ | $2.2\times10^{18}$ | $2.2\times10^{18}$ |
| $N_v(cm^{-3})$ | $1.8\times10^{19}$ | $3.9\times10^{18}$ | $3.9\times10^{18}$ | $3.9\times10^{18}$ | $1.8\times10^{19}$ | $1.8\times10^{19}$ |
| $\mu_e(cm^2/(VS))$ | $2\times10^{-4}$ | 10 | 10 | 10 | 100 | 20 |
| $\mu_h(cm^2/(VS))$ | $2\times10^{-4}$ | 10 | 10 | 10 | 25 | 10 |
| $N_D(cm^{-3})$ | 0 | $1\times10^{9}$ | $1\times10^{9}$ | $1\times10^{9}$ | $1\times10^{19}$ | $2\times10^{19}$ |
| $N_A(cm^{-3})$ | $2\times10^{18}$ | $1\times10^{9}$ | $1\times10^{9}$ | $1\times10^{9}$ | 0 | 0 |
| $N_T(cm^{-3})$ | $1\times10^{15}$ | $2\times10^{16}$ | $2\times10^{15}$ | $3\times10^{15}$ | $1\times10^{15}$ | $1\times10^{15}$ |
| Reference | [1,2] | [2-5] | [2-5] | [2-5] | [2,6] | [7] |

## SC. Initial data for PSC simulation

Table S2–S5 reveal the input parameters for all layers. The thickness of all ETL and HTL is 0.05μm, and the defect density is $1\times10^{15}cm^{-3}$.

Table S2. Input parameters of the absorber layer and FTO.

| Parameters | $Cs_4CuSb_2Cl_{12}$ | $Cs_2TiI_6$ | FTO |
|---|---|---|---|
| Thickness(μm) | 0.65 | 0.65 | 0.1 |
| $E_g$(eV) | 1.6 | 1.8 | 3.5 |
| χ(eV) | 3.74 | 3.98 | 4 |
| $\varepsilon_r$ | 10 | 18 | 9 |
| $N_c(cm^{-3})$ | $1.6\times10^{18}$ | $1\times10^{19}$ | $2.2\times10^{18}$ |
| $N_v(cm^{-3})$ | $4.5\times10^{18}$ | $1\times10^{19}$ | $1.8\times10^{19}$ |
| $\mu_e(cm^2/(VS))$ | 2.5 | 4.4 | 20 |

| | | | |
|---|---|---|---|
| $\mu_h$(cm$^2$/(VS)) | 2.5 | 2.5 | 10 |
| $N_D$(cm$^{-3}$) | 1×10$^{13}$ | 1×10$^{16}$ | 2×10$^{19}$ |
| $N_A$(cm$^{-3}$) | 1×10$^{13}$ | 1×10$^{16}$ | 0 |
| $N_T$(cm$^{-3}$) | 1×10$^{15}$ | 1×10$^{15}$ | 1×10$^{15}$ |
| Reference | [8] | [9] | [7] |

Table S3. Input parameters of interface.

| Parameter | HTL/ CCSC | CCSC/ CTI | CTI/ ETL | ETL/ FTO |
|---|---|---|---|---|
| Defeat type | Neutral | Neutral | Neutral | Neutral |
| $\sigma_e$(cm$^{-2}$) | 1×10$^{-19}$ | 1×10$^{-19}$ | 1×10$^{-19}$ | 1×10$^{-19}$ |
| $\sigma_h$(cm$^{-2}$) | 1×10$^{-19}$ | 1×10$^{-19}$ | 1×10$^{-19}$ | 1×10$^{-19}$ |
| Er(eV) | 0.6 | 0.6 | 0.6 | 0.6 |
| Energy level state dispersion | Single | Single | Single | Single |
| Defeat density(cm$^{-3}$) | 1×10$^{14}$ | 1×10$^{14}$ | 1×10$^{14}$ | 1×10$^{14}$ |
| Reference | [10] | [10] | [10] | [10] |

Table S4. Input parameters of ETL.

| Parameters | $E_g$ (eV) | $\chi$ (eV) | $\varepsilon_r$ | $N_c$ (cm$^{-3}$) | $N_v$ (cm$^{-3}$) | $\mu_e$ (cm$^2$/(VS)) | $\mu_h$ (cm$^2$/(VS)) | $N_D$ (cm$^{-3}$) | References |
|---|---|---|---|---|---|---|---|---|---|
| $BaSnO_3$ | 3.16 | 3.9 | 17 | 1.2×10$^{19}$ | 1.8×10$^{19}$ | 200 | 25 | 1×10$^{18}$ | [11] |
| IGZO | 3.05 | 4.16 | 10 | 5×10$^{18}$ | 5×10$^{18}$ | 15 | 0.1 | 1×10$^{18}$ | [12] |
| LBSO | 3.12 | 4.4 | 22 | 1×10$^{18}$ | 2.2×10$^{20}$ | 70 | 70 | 4.45×10$^{20}$ | [13] |
| PC61PM | 2.1 | 4 | 18 | 2.2×10$^{18}$ | 1.8×10$^{19}$ | 2×10$^{-3}$ | 2×10$^{-3}$ | 1×10$^{18}$ | [14] |
| PCBM | 2.1 | 3.9 | 3.9 | 2.2×10$^{19}$ | 2.2×10$^{19}$ | 1×10$^{-3}$ | 2×10$^{-3}$ | 1×10$^{20}$ | [15] |
| $SnO_2$ | 3.6 | 4.5 | 9 | 2.2×10$^{18}$ | 1.8×10$^{19}$ | 100 | 25 | 1×10$^{20}$ | [6] |
| $SnS_2$ | 1.85 | 4.26 | 17.7 | 7.3×10$^{18}$ | 1×10$^{19}$ | 25 | 50 | 1×10$^{20}$ | [16] |
| STO | 3.2 | 4 | 8.7 | 1.7×10$^{19}$ | 2×10$^{20}$ | 5.3×10$^3$ | 6.6×10$^2$ | 2×10$^{18}$ | [17] |
| $TiO_2$ | 3.26 | 4.2 | 10 | 2.2×10$^{18}$ | 1.8×10$^{19}$ | 100 | 25 | 1×10$^{19}$ | [6] |
| $WS_2$ | 1.8 | 4.3 | 13.6 | 1×10$^{18}$ | 2.4×10$^{19}$ | 100 | 51 | 1×10$^{15}$ | [18] |

| | | | | | | | | | |
|---|---|---|---|---|---|---|---|---|---|
| $Zn_2SO_4$ | 3 | 4.2 | 10 | $1\times10^{18}$ | $1\times10^{18}$ | 16 | 16 | $3.2\times10^{15}$ | [19] |
| ZnS | 2.7 | 4.3 | 10 | $2.2\times10^{18}$ | $1.8\times10^{19}$ | 1 | 4.7 | $9.3\times10^{15}$ | [20] |
| $ZnSnN_2$ | 1.5 | 4.1 | 15 | $1.2\times10^{18}$ | $7.8\times10^{19}$ | $5\times10^{-1}$ | $5\times10^{-2}$ | $1\times10^{19}$ | [21] |

Table S5. Input parameters of HTL.

| Parameters | $E_g$ (eV) | $\chi$ (eV) | $\varepsilon_r$ | $N_c$ ($cm^{-3}$) | $N_v$ ($cm^{-3}$) | $\mu_e$ ($cm^2$/(VS)) | $\mu_h$ ($cm^2$/(VS)) | $N_D$ ($cm^{-3}$) | References |
|---|---|---|---|---|---|---|---|---|---|
| Spiro-OMeTAD | 3 | 2.45 | 3 | $2.2\times10^{18}$ | $1.8\times10^{19}$ | $2\times10^{-4}$ | $2\times10^{-4}$ | $2\times10^{18}$ | [1] |
| CBTS | 1.9 | 3.6 | 5.4 | $2.2\times10^{19}$ | $1\times10^{19}$ | 30 | 10 | $1\times10^{19}$ | [22] |
| CNTS | 1.74 | 3.87 | 9 | $2.2\times10^{19}$ | $1.8\times10^{19}$ | 11 | 11 | $1\times10^{16}$ | [22] |
| $Cu_2O$ | 2.17 | 3.2 | 7.11 | $1.8\times10^{19}$ | $1.8\times10^{19}$ | 20 | 80 | $1\times10^{21}$ | [6] |
| $Cu_2Te$ | 1.18 | 4.2 | 10 | $7.8\times10^{17}$ | $1.6\times10^{19}$ | 500 | 100 | $1\times10^{21}$ | [23] |
| $MoTe_2$ | 1.1 | 4.2 | 13 | $1\times10^{15}$ | $1\times10^{17}$ | 110 | 426 | $5\times10^{18}$ | [24] |
| NiO | 3.8 | 1.46 | 10.7 | $2.8\times10^{19}$ | $1\times10^{19}$ | 120 | 28 | $1\times10^{18}$ | [22] |
| $SrCu_2O_2$ | 3.3 | 2.2 | 9.77 | $2.2\times10^{18}$ | $1\times10^{19}$ | $1\times10^{-1}$ | $4.6\times10^{-1}$ | $6.1\times10^{17}$ | [6] |
| $Zn_2P_3$ | 1.5 | 4.2 | 7.11 | $2.2\times10^{18}$ | $2.2\times10^{18}$ | 1 | 3.8 | $1\times10^{19}$ | [25] |
| ZnTe | 2.25 | 3.73 | 7.3 | $2.2\times10^{18}$ | $1\times10^{19}$ | 300 | 100 | $2\times10^{18}$ | [26] |
| CuSCN | 3.6 | 1.7 | 10 | $2.2\times10^{19}$ | $1.8\times10^{18}$ | 100 | 25 | $1\times10^{18}$ | [27] |
| $CuSbS_2$ | 1.58 | 4.05 | 14.6 | $2.2\times10^{18}$ | $2.2\times10^{19}$ | 49 | 49 | $1\times10^{18}$ | [18] |
| SnS | 1.32 | 4.2 | 14 | $8.9\times10^{18}$ | $4.6\times10^{19}$ | 11.2 | 11.2 | $1\times10^{18}$ | [28] |

# SD. The results of the SHAP algorithm for $V_{oc}$, $J_{sc}$ and FF

The results are shown in Fig. S1–S3.

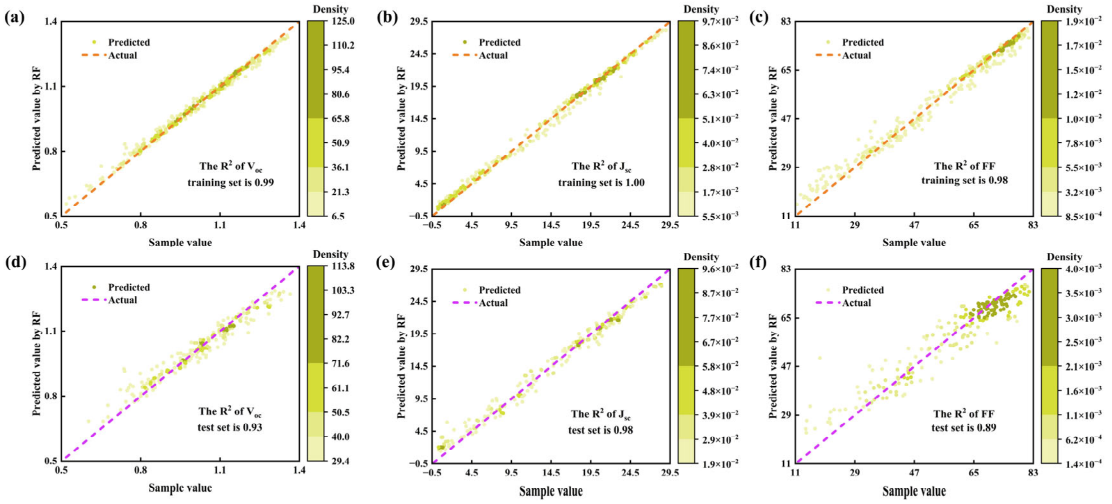


Figure S1. The fitting situation of LR for $V_{oc}$, $J_{sc}$ and FF: (a) The fitting situation of $V_{oc}$ training data; (b) The fitting situation of $V_{oc}$ test data; (c) The fitting situation of $J_{sc}$ training data; (d) The fitting situation of $J_{sc}$ test data; (e) The fitting situation of FF training data; (f) The fitting situation of FF test data.

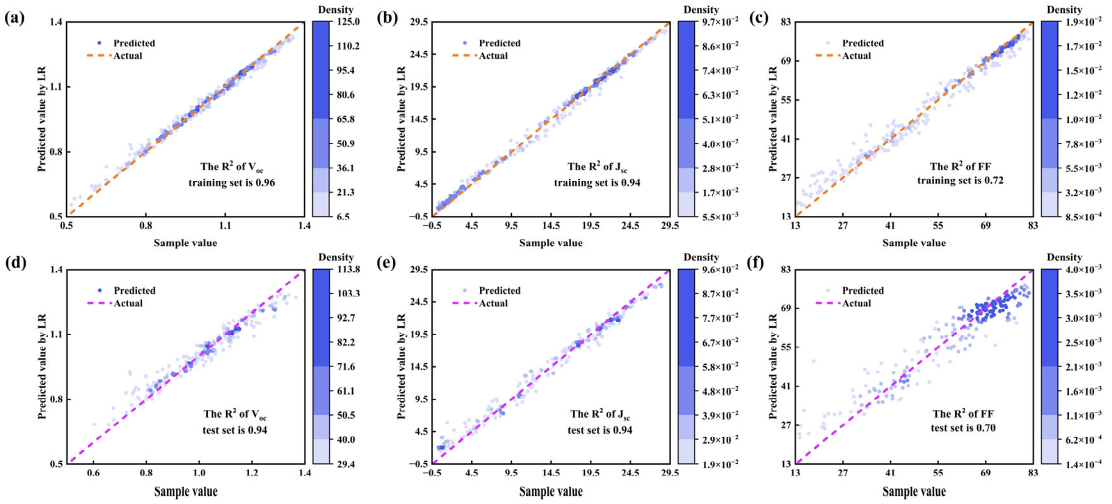


Figure S2. The fitting situation of RF for $V_{oc}$, $J_{sc}$ and FF: (a) The fitting situation of $V_{oc}$ training data; (b) The fitting situation of $V_{oc}$ test data; (c) The fitting situation of $J_{sc}$ training data; (d) The fitting situation of $J_{sc}$ test data; (e) The fitting situation of FF training data; (f) The fitting situation of FF test data.

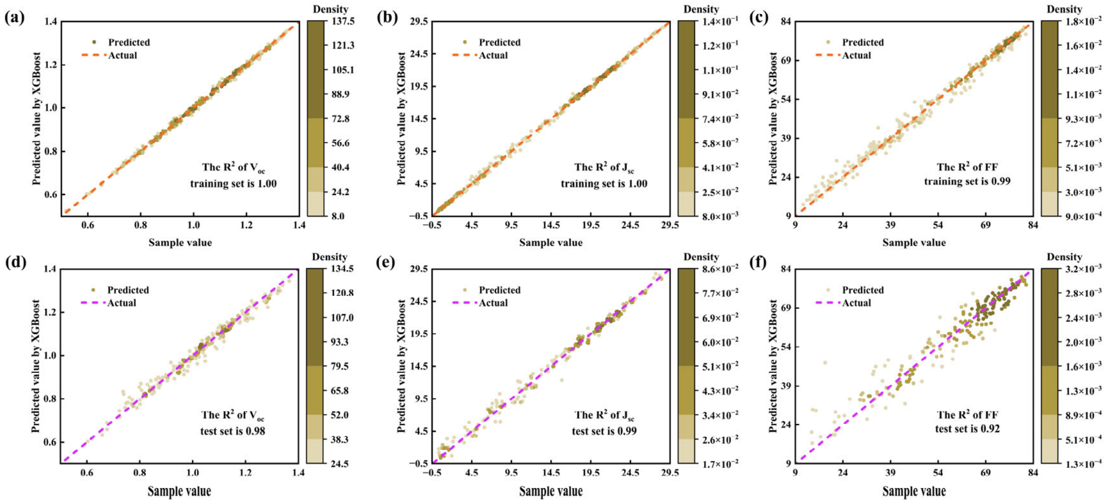


Figure S3. The fitting situation of XGBoost for $V_{oc}$, $J_{sc}$ and FF: (a) The fitting situation of $V_{oc}$ training data; (b) The fitting situation of $V_{oc}$ test data; (c) The fitting situation of $J_{sc}$ training data; (d) The fitting situation of $J_{sc}$ test data; (e) The fitting situation of FF training data; (f) The fitting situation of FF test data.

## SE. Comparison between this study and other studies

Fig. 13(c) in the main text has shown the structure and other information of other studies, and the references are given in Table S6.

Table S6. Comparisons with other studies.

| Equipment composition | Type | PCE | Reference |
|---|---|---|---|
| FTO/IGZO/CCSC/CuO/Au | Theoretical | 17.39% | [29] |
| FTO/$TiO_2$/CCSCNCs/$Cu_2O$/Au | Theoretical | 23.07% | [30] |
| FTO/$WS_2$/$Cs_4CuSb_2Cl_{12}$/$CuSbS_2$/Ni | Theoretical | 23.10% | [31] |
| FTO/$TiO_2$/$Cs_2TiBr_6$/P3HT/Au | Experimental | 1.80% | [32] |
| FTO/$TiO_2$/CTI/P3HT/Au | Experimental | 2.04% | [32] |
| FTO/$TiO_2$/$Cs_2TiCl_6$/P3HT/Au | Experimental | 2.32% | [32] |
| FTO/$TiO_2$/C60/$Cs_2TiBr_6$/P3HT/Au | Experimental | 3.3% | [33] |
| Si/CdS/CTI/CuSCN | Theoretical | 3.13% | [34] |
| ITO/Nb2O5/CTI/PEDOT:PSS/Au | Theoretical | 18.5% | [35] |
| TCO/ZnO/CTI/CdTe/Au | Theoretical | 23.51% | [36] |
| FTO/STO/CTI/CCSC/CuSCN/Cu-doping C | Theoretical | 26.60% | This work |

## Reference


[1] A.A. Kanoun, M.B. Kanoun, A.E. Merad, S. Goumri-Said, Toward development of high-performance perovskite solar cells based on $CH_3NH_3GeI_3$ using computational approach, Sol. Energy. 182 (2019) 237-244. https://doi.org/10.1016/j.solener.2019.02.041.

[2] M.Z. Liu, M.B. Johnston, H.J. Snaith, Efficient planar heterojunction perovskite solar cells by vapour deposition, Nature. 501 (2013) 395-+. https://doi.org/10.1038/nature12509.

[3] C.C. Homes, T. Vogt, S.M. Shapiro, S. Wakimoto, A.P. Ramirez, Optical response of high-dielectric-constant perovskite-related oxide, Science. 293 (2001) 673-676. https://doi.org/10.1126/science.1061655.

[4] C. Wehrenfennig, G.E. Eperon, M.B. Johnston, H.J. Snaith, L.M. Herz, High Charge Carrier Mobilities and Lifetimes in Organolead Trihalide Perovskites, Adv. Mater. 26 (2014) 1584-1589. https://doi.org/10.1002/adma.201305172.

[5] G. Giorgi, J.I. Fujisawa, H. Segawa, K. Yamashita, Small Photocarrier Effective Masses Featuring Ambipolar Transport in Methylammonium Lead Iodide Perovskite: A Density Functional Analysis, J. Phys. Chem. Lett. 4 (2013) 4213-4216. https://doi.org/10.1021/jz4023865.

[6] M. Shasti, A. Mortezaali, Numerical Study of $Cu_2O$, $SrCu_2O_2$, and $CuAlO_2$ as Hole-Transport Materials for Application in Perovskite Solar Cells, Phys. Status Solidi A-Appl. Mat. 216 (2019) 10. https://doi.org/10.1002/pssa.201900337.

[7] S. Ali, P. Kumar, K. Ahmad, R.A. Khan, Simulation of Lead-Free Perovskite Solar Cells with Improved Performance, Crystals. 15 (2025) 12. https://doi.org/10.3390/cryst15020171.

[8] S. Tahir, S. Mushtaq, R. Saeed, J. Iqbal, M.D. Alshahrani, R.S. Almufarij, I. Ragab, A. Ashfaq, Numerical simulation of $Cs_2AgInBr_6$/$Cs_4CuSb_2Cl_{12}$ heterojunction perovskite solar cell: A path to achieve optimized performance, Physica B. 703 (2025) 13. https://doi.org/10.1016/j.physb.2025.417011.

[9] M.A. Islam, M.M. Haque, V. Selvanathan, M. Mottakin, D.K. Sarkar, K. Joya, A.M. Alanazi, T. Suemasu, I.M. Syed, M. Akhtaruzzaman, Theoretical Analysis on Interfacial Dynamics Between Charge Transport Layer and Different Absorbers in Pb-free All Inorganic Perovskites Solar Cells, J. Electron. Mater. 53 (2024) 6823-6837. https://doi.org/10.1007/s11664-024-11372-7.

[10] S. Bimli, V. Manjunath, S.R. Mulani, A. Miglani, O.S. Game, R.S. Devan, Theoretical investigations of all inorganic $Cs_2SnI_6$ double perovskite solar cells for efficiency ~ 30 %, Sol. Energy. 256 (2023) 76-87. https://doi.org/10.1016/j.solener.2023.03.059.

[11] A. Tara, V. Bharti, H. Dixit, S. Sharma, R. Gupta, Performance evaluation of all-inorganic cesium-based perovskite solar cell with $BaSnO_3$ as ETL, J. Nanopart. Res. 25 (2023) 14. https://doi.org/10.1007/s11051-023-05830-2.

[12] F. Azri, A. Meftah, N. Sengouga, A. Meftah, Electron and hole transport layers optimization by numerical simulation of a perovskite solar cell, Sol. Energy. 181 (2019) 372-378. https://doi.org/10.1016/j.solener.2019.02.017.

[13] M.K. Hossain, O. Alsalman, S. Rana, M.S. Uddin, G.F.I. Toki, S.H. Shahatha, M.R. Mohammad, M.A. Darwish, P. Sasikumar, S. Haq, H. Bencherif, R. Haldhar, Enhancing efficiency and performance of $Cs_2TiI_6$-based perovskite solar cells through extensive optimization: A numerical approach, Inorg. Chem. Commun. 168 (2024) 13. https://doi.org/10.1016/j.inoche.2024.112964.

[14] T. AlZoubi, B. Mourched, M. Al Gharram, G. Makhadmeh, O. Abu Noqta, Improving Photovoltaic Performance of Hybrid Organic-Inorganic $MAGeI_3$ Perovskite Solar Cells via Numerical Optimization of Carrier Transport Materials (HTLs/ETLs), Nanomaterials. 13 (2023) 17. https://doi.org/10.3390/nano13152221.

[15] U. Mandadapu, V. Vedanayakam, K. Thyagarajan, Simulation and Analysis of Lead based Perovskite Solar Cell using SCAPS-1D, Ind. J. Sci. Technol. 10 (2017) 1-8. https://doi.org/10.17485/ijst/2017/v10i11/110721.

[16] A. Rahmoune, O. Babahani, Numerical analysis of Al/Gr/ETL/$MoS_2$/$Sb_2S_3$/Ni solar cell using non-toxic $In_2S_3$/$SnS_2$/ZnSe electron transport layer, Optik. 283 (2023) 20. https://doi.org/10.1016/j.ijleo.2023.170875.

[17] S. Bishnoi, S.K. Pandey, Device performance analysis for lead-free perovskite solar cell optimisation, IET Optoelectron. 12 (2018) 185-190. https://doi.org/10.1049/iet-opt.2017.0135.

[18] S. Mukaddar, Optimization of efficiency of $CsPbI_2Br$ by using different electron transport and hole transport layers: A DFT and SCAPS-1D simulation, Micro Nanostructures. 197 (2025) 17. https://doi.org/10.1016/j.micrna.2024.208024.

[19] M.A. Matin, M.M. Aliyu, A.H. Quadery, N. Amin, Prospects of novel front and back contacts for high efficiency cadmium telluride thin film solar cells from numerical analysis, Sol. Energy Mater. Sol. Cells. 94 (2010) 1496-1500. https://doi.org/10.1016/j.solmat.2010.02.042.

[20] S. Jamal, A.D. Khan, A.D. Khan, High performance perovskite solar cell based on efficient materials for electron and hole transport layers, Optik. 218 (2020) 6. https://doi.org/10.1016/j.ijleo.2020.164787.

[21] A. Laidouci, A. Aissat, J.P. Vilcot, Numerical study of solar cells based on $ZnSnN_2$ structure, Sol. Energy. 211 (2020) 237-243. https://doi.org/10.1016/j.solener.2020.09.025.

[22] Y.H. Khattak, F. Baig, H. Toura, S. Beg, B.M. Soucase, CZTSe Kesterite as an Alternative Hole Transport Layer for $MASnI_3$ Perovskite Solar Cells, J. Electron. Mater. 48 (2019) 5723-5733. https://doi.org/10.1007/s11664-019-07374-5.

[23] S.R. Al Ahmed, M. Rahaman, A. Sunny, S. Rahman, M.S. Islam, T.A. Taha, Z.A. Alrowaili, M.S. Mian, Enhancing the efficiency of $Cu_2Te$ thin-film solar cell with $WS_2$ buffer layer: A simulation study, Opt. Laser Technol. 159 (2023) 11. https://doi.org/10.1016/j.optlastec.2022.108942.

[24] A.K. Patel, P.K. Rao, R. Mishra, S.K. Soni, Numerical study of a high-performance thin film CIGS solar cell with a-Si and $MoTe_2$ hole transport layer, Optik. 243 (2021) 10. https://doi.org/10.1016/j.ijleo.2021.167498.
[25] M.Q. Kareem, S.A. Hassan, S.S. Alimardan, S.M. Shareef, M.M. Ameen, CHTS/$Zn_2P_3$-based solar cells with enhanced efficiency through ETL engineering: A numerical study, J. Phys. Chem. Solids. 188 (2024) 12. https://doi.org/10.1016/j.jpcs.2024.111931.
[26] K. Kumari, A. Jana, A. Dey, T. Chakrabarti, S.K. Sarkar, Lead free $CH_3NH_3SnI_3$ based perovskite solar cell using ZnTe nano flowers as hole transport layer, Opt. Mater. 111 (2021) 7. https://doi.org/10.1016/j.optmat.2020.110574.
[27] Y. Raoui, H. Ez-Zahraouy, N. Tahiri, O. El Bounagui, S. Ahmad, S. Kazim, Performance analysis of $MAPbI_3$ based perovskite solar cells employing diverse charge selective contacts: Simulation study, Sol. Energy. 193 (2019) 948-955. https://doi.org/10.1016/j.solener.2019.10.009.
[28] M. Minbashi, A. Ghobadi, M.H. Ehsani, H.R. Dizaji, N. Memarian, Simulation of high efficiency SnS-based solar cells with SCAPS, Sol. Energy. 176 (2018) 520-525. https://doi.org/10.1016/j.solener.2018.10.058.
[29] K.D. Jayan, Bandgap Tuning and Input Parameter Optimization for Lead-Free All-Inorganic Single, Double, and Ternary Perovskite-Based Solar Cells, Sol. RRL. 6 (2022) 15. https://doi.org/10.1002/solr.202100971.
[30] Y.Z. He, L.Y. Xu, C. Yang, X.W. Guo, S.R. Li, Design and Numerical Investigation of a Lead-Free Inorganic Layered Double Perovskite $Cs_4CuSb_2Cl_{12}$ Nanocrystal Solar Cell by SCAPS-1D, Nanomaterials. 11 (2021) 19. https://doi.org/10.3390/nano11092321.
[31] H. Karmaker, A. Siddique, B.K. Das, M.N. Islam, Modeling and performance investigation of novel inorganic $Cs_4CuSb_2Cl_{12}$ nanocrystal perovskite solar cell using SCAPS-1D, Results Eng. 22 (2024) 102106. https://doi.org/https://doi.org/10.1016/j.rineng.2024.102106.
[32] K. Chakraborty, N.R. Medikondu, P.B. Kanakavalli, V.V. Kamesh, S. Das, M.G. Choudhury, S. Paul, Comparative study of structural, opto-electronic properties of $Cs_2TiX_6$-based single halide double perovskite solar cells: computational and experimental approach, Phys. Scr. 99 (2024) 11. https://doi.org/10.1088/1402-4896/ad77fc.
[33] M. Chen, M.G. Ju, A.D. Carl, Y.X. Zong, R.L. Grimm, J.J. Gu, X.C. Zeng, Y.Y. Zhou, N.P. Padture, Cesium Titanium(IV) Bromide Thin Films Based Stable Lead-free Perovskite Solar Cells, Joule. 2 (2018) 558-570. https://doi.org/10.1016/j.joule.2018.01.009.
[34] K. Chakraborty, M.G. Choudhury, S. Paul, Numerical study of $Cs_2TiX_6$ ($X = Br^-$, $I^-$, $F^-$ and $Cl^-$) based perovskite solar cell using SCAPS-1D device simulation, Sol. Energy. 194 (2019) 886-892. https://doi.org/10.1016/j.solener.2019.11.005.
[35] S.A. Moiz, S.A. Albadwani, M.S. Alshaikh, Towards Highly Efficient Cesium Titanium Halide Based Lead-Free Double Perovskites Solar Cell by Optimizing

the Interface Layers, Nanomaterials. 12 (2022) 17. https://doi.org/10.3390/nano12193435.

[36] K. Fatema, Optimizing inorganic double halide ($Cs_2TiI_6$) perovskite solar cell for different hole transport layers using solar cell capacitance software (SCAPS-ID), Mater. Today Commun. 35 (2023) 7. https://doi.org/10.1016/j.mtcomm.2023.105860.
